# The genetic code at the balance point of error and demand

**DOI:** 10.1371/journal.pcbi.1014613

**Published:** 2026-08-11

**Authors:** Yudam Seo, Tsvi Tlusty, Junghyo Jo

**Affiliations:** 1 Department of Science Education, Seoul National University, Seoul, Korea; 2 Department of Physics, Ulsan National Institute of Science and Technology, Ulsan, Korea; 3 Department of Physics Education, Seoul National University, Seoul, Korea; 4 School of Computational Sciences, Korea Institute for Advanced Study, Seoul, Korea; Universite de Lausanne Faculte de biologie et medecine, SWITZERLAND

## Abstract

The origin and organizing principles of the genetic code remain central problems in molecular evolution. The low probability of the natural codon-to-amino acid mapping arising by chance has spurred the hypothesis that its structure is optimized for robustness to mutations and translational errors. For the construction of effective molecular machines, the repertoire of encoded amino acids must also be diverse enough in physicochemical features. Here, we examine whether the standard genetic code can be understood as a near-optimal solution balancing these two objectives: minimizing error load and aligning codon assignments with the naturally occurring amino acid composition. Using simulated annealing, we explore this trade-off across a broad range of parameters. We find that the standard genetic code resides near an optimum in the fitness landscape of possible genetic codes. The degeneracy of the code plays a dual role, minimizing mistranslation errors while matching codon multiplicity to amino acid usage frequencies. As a result, uniform codon usage alone is sufficient to recover the empirical amino acid composition, without any additional bias. It is a highly effective solution that balances fidelity against resource availability constraints. A comparative analysis of natural variants also reveals a functional decoupling: error robustness acts as a rigid global constraint determined by code topology, whereas compositional alignment serves as a more flexible variable that adapts to lineage-specific demands. These results support a multi-objective optimization framework in which the genetic code reflects a balance between translational fidelity and proteomic demand.

## Author summary

The genetic code is the fundamental language of life, translating DNA into proteins, the molecules that perform most cellular functions. In this code, groups of three nucleotides—called codons—act like three-letter words that specify which amino acid will be added to a growing protein. Although many different codon–amino acid assignments are theoretically possible, nearly all organisms share the same “standard” genetic code. Previous studies have shown that this code is unusually robust: mutations or translation errors often cause only small changes in the resulting proteins. However, building living organisms requires more than error tolerance. Cells must also produce proteins using amino acids in proportions that match biological demand. Here we test whether the genetic code reflects a balance between these two pressures: minimizing errors and aligning codon assignments with amino acid usage. Using computational simulations, we explore many possible genetic codes and evaluate their performance under these competing objectives. We find that the standard genetic code lies near optimal solutions balancing translational fidelity and amino acid demand. More broadly, our results illustrate how fundamental biological systems can emerge from evolutionary trade-offs between reliability and functional resource requirements.

## Introduction

The standard genetic code (SGC) is a nearly universal mapping governing the translation of genomic information into functional proteomes across the entire tree of life, with only a few exceptions [1,2]. The combinatorial space formed by 64 codons and 20 amino acids yields an approximately $21!\cdot S(64,21)\sim 10^{84}$ potential mappings (or $\sim 10^{79}$ if stop codons are excluded [3,4]). However, the emergence and near-universal conservation of a single mapping remain incompletely understood [3,4]. This problem is traditionally divided into two interrelated inquiries: (i) the mechanisms of *stabilized universality*, addressing why a single mapping is preserved almost perfectly unlike typical plastic genetic traits, and (ii) the *initial conditions*, concerning whether specific codon assignments within the SGC possess unique and non-random characteristics.

Francis Crick’s *frozen accident theory* [5] speaks chiefly to the first question. Once a substantial proteome had come to depend on a particular dictionary, reassigning any codon would have altered many proteins simultaneously and imposed an intolerable load, effectively locking the code against further change [6,7]. Engineered organisms with recoded genomes or reassigned codons remain viable but pay a measurable fitness cost [8–10], indicating that little further optimization was attainable once the code had frozen. This freezing is logically distinct from Crick’s secondary suggestion that the original assignments were a historical “accident.” On that second question, the markedly non-random organization of the SGC is difficult to reconcile with pure chance [11,12], motivating the selection-based accounts considered below; a comprehensive survey of these competing hypotheses is given by Kun and Radványi [13].

Consistent with the frozen-accident constraint, the documented departures from the SGC are few and conservative. Most reassign stop codons (for instance, UGA to tryptophan in *Mycoplasma*, UAG and UAA to glutamine in ciliates, and UGA to cysteine in *Euplotes*), leaving the amino acid content of existing proteins essentially intact [1,2]. Reassignments between sense codons, which do change protein composition, are rarer; the best-studied example, CUG to serine in the *Candida* CTG clade, appears to have passed through a prolonged stage of ambiguous decoding, again underscoring the cost of altering an established code.

The observation that codons differing by a single base substitution are overwhelmingly assigned to amino acids sharing similar physicochemical properties [14] implies that the code’s architecture is not a mere residue of chance, but rather a product of selection or physical interactions geared toward *translational fidelity*.

In this context, the *stereochemical theory* [15–17] proposes a primitive, direct affinity between amino acids and their cognate codons. Yet, the functional viability of tRNAs with artificially altered anticodons and the low, non-specific binding energies measured between RNA bases and amino acids suggest that a purely physical link did not dictate the final code. Rather, such interactions likely provided an initial bias, shaping the earliest assignments of amino acids to their cognate codons (or proto-tRNA anticodons) during the emergence of the code [18–21].

The *error minimization theory* is premised on the observation that the codon assignment of the SGC efficiently reduces the phenotypic cost of transcriptional and translational errors [22–27]. Quantitative analyses by Haig and Hurst [28] and subsequent statistical studies by Freeland and Hurst [29,30] have demonstrated the improbability of the SGC’s configuration arising by chance, with its superior robustness to errors estimated as a statistical outlier with a probability of roughly “one in a million.” This suggests that the SGC is a highly optimized mapping selected to preserve coherent biological information in the presence of inherent noise arising from molecular imperfections [6,7,31–33].

However, error minimization alone cannot explain the code’s mapping; it must be balanced with the *diversity of the amino acid vocabulary*. A code optimized solely for error tolerance would converge into a degenerate state encoding a single amino acid, lacking the *coding capacity* required to construct complex molecular machines. Such considerations led to the articulation of “physicochemical diversity” measures [34] and the identification of “diversifying steps” along coevolutionary trajectories [12].

In previous works, we integrated the imperative for functional diversity within the information-theoretic framework of the SGC’s emergence and evolution [7,25,35–37]. Yet, these models often relied on simplified, qualitative measures of diversity. The idea that amino acid frequencies themselves carry information about code optimality has a longer history: King and Jukes [38] first noted the correlation between codon degeneracy and the frequency of each amino acid in proteins, and Gilis *et al.* [39] later formalized it by weighting amino acid substitution costs by empirical amino acid frequencies in a fitness function for code optimality, thereby establishing amino acid frequency as a quantitative parameter in such analyses. More recently, Radványi and Kun [40] incorporated organism-specific codon usage into analyses of the code’s mutational robustness. Here, we quantify the joint contributions of translational error load and amino acid compositional alignment by evaluating the genetic code’s fitness against *empirical proteomic demands* [35,39] across 28 diverse phylogenetic lineages. We quantify functional diversity as the compositional alignment between codon-induced supply and the actual amino acid abundances observed in nature [41]. This formulation enables systematic exploration of both the local and global fitness landscape of genetic codes.

Our analysis demonstrates that the SGC’s redundant encoding of highly utilized residues, such as leucine, serine, and arginine, reflects material constraints required for the efficient mass production of cellular machinery. This suggests that the code’s architecture is well matched to the material demands of the proteome it supports. To explore this, we first establish a computational framework by introducing position-dependent mistranslation rates and defining the two primary objective functions of code optimality: (i) translational error load and (ii) amino acid compositional alignment. Using this framework, we demonstrate that the SGC represents a local optimum achieved through the balance of these two selective pressures, followed by a comparative analysis of genetic code variants across diverse taxa. Finally, we discuss the broader evolutionary implications and limitations of our study.

## Materials and methods

### Genomic data collection and processing

To evaluate the evolutionary fitness of the genetic code across diverse biological contexts, we assembled a curated panel of 28 species. Rather than maximizing the number of taxa, we balanced the panel to span the tree of life while limiting the over-representation of any single clade, comprising 12 bacteria, 5 archaea, and 11 eukaryotes. Bacterial and archaeal members were selected to follow recent genome-based phylogenies [42,43], and, given evidence that the SGC froze in a temperate environment [44], mesophilic genomes constitute a clear plurality of the prokaryotic set, with a few thermophiles and halophiles retained to test robustness to environmental extremes. The panel spans eight NCBI genetic code types [45]: the standard code (SGC, type 1); the bacterial and archaeal code (type 11), which shares the SGC’s amino acid assignments and differs only in start-codon usage; and six variant codes (types 4, 6, 10, 12, 25, and 26), comprising both bacterial variants (types 4 and 25) and eukaryotic ones (types 6, 10, 12, and 26). Including these variants lets us test whether error minimization and compositional alignment hold beyond the universal code.

For each species we estimated the proteomic demand for amino acid α, denoted $f_{\alpha }$, as its usage frequency at the protein level. For 13 of the 28 species we computed $f_{\alpha }$ as the abundance-weighted amino acid frequency over the UniProt reference proteome, using per-protein abundances from the PaxDb integrated dataset [46], so that highly expressed proteins contribute in proportion to their cellular abundance. For 12 species without integrated abundance data we used the unweighted UniProt reference proteome, and for the remaining 3 species, where no curated proteome was available, we fell back to translating NCBI coding sequences (CDS). The data source for each species is recorded in the “Source” column of S1 Table, alongside its taxonomy ID, assembly level, and sequence counts, so that the results can be stratified by data quality.

Estimating $f_{\alpha }$ from measured protein abundances, rather than from coding sequences, substantially weakens a circularity present in the analysis. When $f_{\alpha }$ is tallied from CDS, it is constrained by the same codon composition that defines the supply $p_{\alpha }$, so part of any apparent match between demand and supply could be arithmetic rather than evolutionary. Protein abundances are measured independently of the codon table, by mass spectrometry, and therefore decouple $f_{\alpha }$ from $p_{\alpha }$. Because the genetic code mediates all protein synthesis, this circularity cannot be eliminated entirely; we show below that our conclusions are robust to the choice of data source, and we revisit the issue in the Discussion.

### Position-dependent mistranslation dynamics

The translation of genetic information is governed by the mapping of 64 nucleotide triplets (codons) to amino acids, and the rate at which one codon is misread as another during decoding is not uniform. Following Freeland and Hurst [29], who derived their per-position weights from reported mistranslation biases and motivated the transition/transversion weighting by the substitution bias shared by mutation and misreading, we treat these misreading rates as set by the biochemical nature of codon–anticodon mismatches and by their position within the codon triplet.

#### 1. Substitution biases: Transitions vs. transversions.

Misreadings are classified by the structural relationship between the intended base and the one mistakenly paired. *Transition-type* errors keep the substitution within a structural class (purine–purine, A↔G; pyrimidine–pyrimidine, C↔U), whereas *transversion-type* errors cross classes (purine↔pyrimidine). A transition-type misreading produces a purine–pyrimidine codon–anticodon mismatch, most notably the G·U wobble pair, whose near-Watson–Crick geometry is readily accommodated within the ribosomal decoding center; transversion-type errors instead require purine–purine or pyrimidine–pyrimidine mismatches, whose distorted geometry is rejected more efficiently. Transition-type errors therefore tend to dominate near-cognate misreading [47]. The same transition bias is well documented for point mutations, which share these base-pairing preferences [48]. Because the SGC largely assigns codons related by transitions to identical or physicochemically similar amino acids, the prevalence of transition-type errors translates into predominantly conservative substitutions, reinforcing the code’s error-minimizing organization [14].

#### 2. Position-dependent selective constraints.

The functional impact of an error is highly sensitive to its position within the codon:

- **First position**: The first base of a codon plays an important role in determining the amino acid specified. Only leucine (Leu), arginine (Arg), and serine (Ser) have synonymous codons that differ at the first base.
- **Second position**: The second base of a codon is also crucial in determining the amino acid specified. Only serine (Ser) has synonymous codons that differ at the second base.
- **Third (Wobble) position**: Most of the redundancy in the SGC is attributable to the third base of a codon. This position is highly robust against transitions. Such robustness is reflected in the fact that third-position transitions are all synonymous, except for AUA (isoleucine) changing to AUG (methionine, start codon) and UGA (stop codon) changing to UGG (tryptophan). In contrast, third-position transversions, although less disruptive than errors at the first or second positions, are considerably more likely to change the encoded amino acid than transitions.

Codon-resolved measurements of the transition bias specific to mistranslation are scarce, but the same qualitative bias is well characterized for point mutation, with which it shares the base-pairing origins described above. The transition-to-transversion ratio γ≡ti/tv exceeds the value of 0.5 expected under unbiased substitution in essentially all lineages [49]: it is approximately 2.5 in *Arabidopsis thaliana* [50] and around 2.0 genome-wide in humans, rising to approximately 3.0 within exome data [51,52]. We invoke these values as a proxy indicating that transitions are strongly favored, which motivates the range of transition weights explored below. This borrowing is principled rather than circular: the bias toward transition-type mismatches reflects a base-pairing geometry partly shared between replication and decoding, so the mutational γ guides the plausible range of $w_{tt}$ without implying that the two processes occur at equal rates.

The third codon position is also the most error-prone site during decoding, because wobble pairing there is geometrically permissive and misreadings concentrate accordingly. The mutational landscape shows a parallel pattern: the composition of the third position varies far more with genomic GC content (sensitivities of roughly 31:12:80 for the first, second, and third positions) than that of the first or second, marking it as the least constrained and most variable site [53]. We therefore order the per-position susceptibility as third > first > second, consistent with the position weighting that Freeland and Hurst found necessary to capture the SGC’s non-random organization [29,30].

Under the assumption of position-wise independence, the probability of a codon *XYZ* being misread as *xyz* is given by:


$$\begin{array}{c} \mu_{\left(XYZ\to xyz\right)}=P(X\to x)\times P(Y\to y)\times P(Z\to z), \end{array}$$ (1)

where the elements of matrix *P* are defined by the transition and transversion weights at each site. The weights used in Freeland and Hurst’s simulation model [29] are given in Table 1.

**Table 1:** The mistranslation weights used in the prior study (Freeland & Hurst, 1998).

	1st position	2nd position	3rd position
For transitions	1	0.5	1
For transversions	0.5	0.1	1

Based on these weights, the aggregate misreading rates for each codon position (calculated as $w_{i}^{ti}+2\times w_{i}^{tv}$) exhibit a ratio of P1:P2:P3=2:0.7:3. While the P1:P2 ratio aligns with the observed GC-content slopes, the weight assigned to the third position appears underestimated relative to its empirical lability. Although GC-slope ratios and error parameters are not strictly equivalent, the comparison suggests that the third position is considerably more error-prone in natural systems, an inference reinforced by wobble pairing, which is localized to the third position and inherently permits greater variation. Moreover, the weights in Table 1 yield an effective γ≈0.78, far below the transition bias reported for eukaryotes such as humans and *Arabidopsis thaliana* [50,52]. We therefore increased the third-position transition weight, $w_{tt}\equiv w_{3}^{ti}$, to better reflect the empirical transition bias (Table 2).

**Table 2:** The mistranslation weights used in this study.

	1st position	2nd position	3rd position
For transitions	1	0.5	$w_{tt}$
For transversions	0.5	0.1	1

Based on the new weights in Table 2, $w_{tt}$ evaluates to 4.9 and 8.1 for empirical γ values of 2.0 and 3.0, respectively. Since the transition-to-transversion ratio is not universally conserved and fluctuates significantly across diverse taxa and even within specific genomic regions [54], defining a singular, fixed value for $w_{tt}$ is impractical. However, the SGC’s inherent structural resilience suggests a clear biological requirement for transitional stability at the third position. Therefore, to ensure analytical robustness and account for this natural variability, our subsequent statistical assessments are conducted over the parametric range $w_{tt}\geq 1$.

### Metrics for code optimality

To evaluate the evolutionary fitness of the genetic code, we developed a performance metric that integrates translational fidelity with functional diversity. This framework accounts for the asymmetric costs of errors and the requirement for a balanced amino acid repertoire.

#### Translational error load.

To evaluate the error-minimizing properties of the genetic code, it is imperative to establish a quantitative metric for physicochemical distances between amino acids. Following foundational studies on the code’s robustness to decoding errors [23,29,30,55,56], we define amino acid proximity based on *polar requirements* (ϕ). This metric is derived from the experimental chromatographic behavior of amino acids in dimethylpyridine (DMP) solutions [14,57], expressed as:


$$\begin{array}{c} \phi =-\left[\frac{d\left(\ln R_{m}\right)}{d\left(\ln\chi_{w}\right)}\right],\;R_{m}=\frac{1-R_{f}}{R_{f}}=\frac{\nu_{solvent}-\nu_{sample}}{\nu_{sample}}, \end{array}$$ (2)

where $R_{f}$ represents the retardation factor, $\nu_{\text{sample}}$ denotes the migration distance of the solute, and $\nu_{\text{solvent}}$ is the distance traveled by the solvent front. Essentially, ϕ corresponds to the negative slope of a log-log plot of the $R_{m}$ value against the water mole fraction ($\chi_{w}$). These distinct slopes reflect specific molecular interactions between organic bases and amino acid residues; during solvation, both polar and nonpolar forces contribute. Stronger nonpolar interactions reduce the relative demand for polar stabilization. Consequently, amino acids with bulky aliphatic side chains, such as leucine, exhibit shallower slopes and lower ϕ values compared to smaller residues like alanine [14].

**Fig 1:**
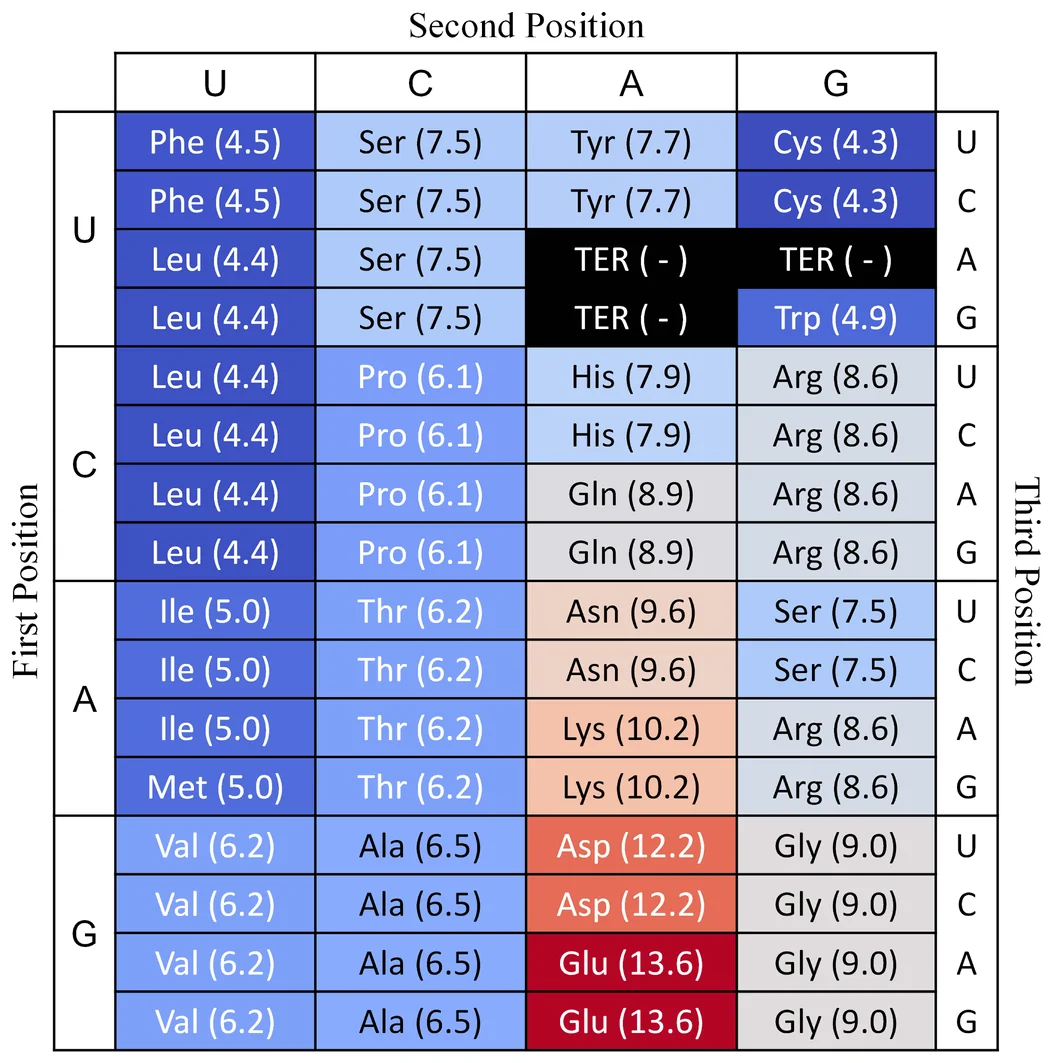
Visualization of the standard genetic code (SGC) mapped by amino acid polar requirement (ϕ). Colors range from blue (low ϕ) to red (high ϕ), with the specific numerical values for each amino acid indicated in parentheses. The spatial clustering of similar colors highlights the non-random, robust architecture of the code, where single-base neighbors tend to specify residues with closely matched physicochemical properties, thereby minimizing the impact of translational errors.

The pattern of the SGC shown in Fig 1 suggests that codon assignments are highly correlated with the values of polar requirements. Based on this characteristic, the error load of the genetic code can be defined in terms of differences in polar requirement, denoted as |Δϕ|. Previous studies assessing the error minimization of the genetic code have typically used absolute error [23] or the squared error [29,55,56] of polar requirement differences [31]. This concept can be generalized such that for a positive real number *n* [32], the error load takes the form:


$$\begin{array}{c} E=\sum_{i,j}\mu_{ij}|\phi_{i}-\phi_{j}{|}^{n}. \end{array}$$ (3)

Here, $\mu_{ij}$ denotes the misreading probability between codons *i* and *j*, derived from the position-dependent weights *w*. For instance, consider the misreading of UUC as AUU. Assuming a per-codon misreading probability *c*, this specific path involves a first-position transversion and a third-position transition. Applying the baseline weights from Table 1, $\mu_{ij}$ is calculated as $w_{1}^{\text{tv}}c\times (1-w_{2}^{\text{ti}}c-2w_{2}^{\text{tv}}c)\times w_{3}^{\text{ti}}c=0.5c^{2}-0.35c^{3}$. To maintain comparability while varying *w*_tt_, each weight is normalized by $k=\sum w_{i}=4.7+w_{tt}$, which equals 5.7 for the Table 1 baseline ($w_{tt}=1$). Previous simulations using *n* = 2 (squared error) revealed that only one in every million random codes is more efficient than the SGC, underscoring the code’s exceptional optimization for error robustness [29,30].

While the squared error is an empirically validated metric, the code’s optimality remains robust across various values of *n* [32] and alternative distance measures, such as the Grantham [58], Miyata [59], EMPAR [60], and other matrices [61,62]. We confirm this directly within our framework: across the random-code ensemble, the natural codes remain pronounced low-error outliers not only under polar requirement but also under hydropathy [63] and molecular volume [58], with a weaker but still clearly significant signal under isoelectric point (S2 Appendix). However, sequence-derived matrices (e.g., PAM [31,64]) are inherently contingent on the existing SGC, potentially introducing circularity into evolutionary models. Physicochemically defined metrics like ϕ are independent of the current code architecture and therefore provide an unbiased framework for modeling the prebiotic or early evolutionary landscape of the SGC [65]. This independence is particularly relevant given that amino acid exchangeabilities can vary across taxa and lineages.

**Fig 2:**
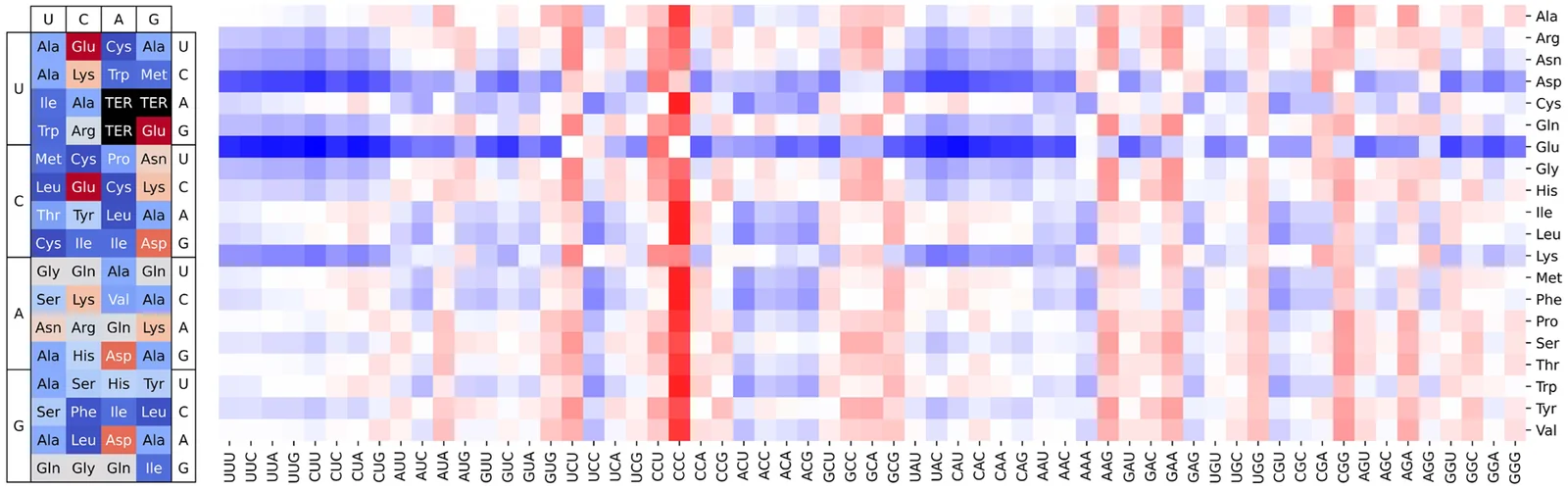
Analysis of local variation for a randomly generated genetic code. Left: The random code table mapped by polar requirement (ϕ), following the visualization format of Fig 1. Unlike the SGC, this code lacks coherent spatial clustering of physicochemical properties. Right: Heatmap representing the change in error load resulting from single-codon reassignments. Each column corresponds to a codon κ subjected to variation, while each row represents the newly assigned amino acid. Red cells indicate a reduction in error load (improvement), whereas blue cells indicate an increase (deterioration). For example, the codon CCC is assigned to glutamic acid (Glu), which has significantly different polar requirements compared with its single-base neighbors (especially third-position transitions). Consequently, most reassignments at CCC result in reduced error loads, as shown by distinct vertical red bands. This heatmap contains all 61×19=1,159 possible neighboring codes.

To investigate whether the SGC resides at a local optimum, we employ local variation analysis to explore its fitness landscape. A neighboring code is defined as a variant differing from the SGC by exactly one codon-to-amino acid reassignment. By systematically examining all such single-step deviations (Fig 2), we (i) identify the optimal sensitivity parameter *n* that best characterizes error minimization in the polar requirement metric, and (ii) assess whether the SGC represents a local optimum under various misreading-weight biases.

As previously established, empirical γ values typically exceed 1.5. In regimes of elevated $w_{tt}$, a genetic code can achieve optimality only if synonymous codons connected by third-position transitions are structurally clustered to buffer against these dominant errors. Specifically, for the SGC to be identified as a local optimum through incremental amino acid substitutions, its optimality should be attainable at biologically plausible values of $w_{tt}$, provided a ‘natural’ error metric is employed. Consequently, we evaluate the suitability of the genetic code using squared error, absolute error, and the generalized *n*-power error metric, by exploring local variation around the SGC and gradually increasing the value of $w_{tt}$ from unity, to determine the minimal point at which the code becomes locally optimal.

#### Amino acid compositional alignment.

The error load of a genetic code is fundamentally constrained by its codon-adjacency topology [7,36,66] and the physicochemical distances between encoded residues. Yet, a model driven solely by error minimization leads to a degenerate global optimum in which all codons specify a single amino acid (Δϕ=0,E=0), a biologically non-functional state. For organismal survival, the code must maintain a diverse repertoire of 20 amino acids. Furthermore, the non-uniform allocation of codons observed in nature likely reflects an adaptive strategy to satisfy specific proteomic demands while buffering against stochastic errors. The relative frequencies of amino acids, therefore, must be integrated into any robust evaluation of code optimality.

Incorporating this logic into our framework provides insight into the non-uniform codon assignments observed in nature. While previous studies considered the diversity of the amino acid vocabulary using simplified or qualitative measures of diversity [36,37], here we explicitly account for the nuanced distribution of amino acids observed in present-day organisms. This consideration motivates the introduction of an amino acid Kullback–Leibler divergence (KLD) term to quantify the evolutionary pressures exerted by varying amino acid demands.

To account for the non-uniform proteomic demand for each amino acid during protein synthesis, we define the amino acid KLD term as follows:


$$\begin{array}{c} D=D_{KL}\left(f_{\alpha }∥p_{\alpha }\right)=\sum_{\alpha \in AA}f_{\alpha }\ln\frac{f_{\alpha }}{p_{\alpha }}. \end{array}$$ (4)

Here, $f_{\alpha }$ denotes the frequency of amino acid α∈AA in the proteome, where AA represents the set of 20 canonical amino acids. Whereas previous studies relied on published, organism-independent frequency data [67–71], we estimate $f_{\alpha }$ separately for each organism from protein-level abundance data, as described above, so as to capture lineage-specific demand. We denote by $p_{\alpha }$ the proportion of sense codons assigned to amino acid α in a given code.

To quantify the match between codon allocation and amino acid demand, we employ the KLD. Mathematically, KLD is asymmetric and does not satisfy the triangle inequality required for a true distance metric. While the Jensen–Shannon divergence (JSD) could serve as a symmetric and bounded alternative, we deliberately adopted the forward KLD definition, $D_{\text{KL}}(f_{\alpha }∥p_{\alpha })$, to reflect the inherent directionality of the biological constraint. In our framework, $f_{\alpha }$ represents the “true” target distribution (biological demand) that the genetic code must satisfy, whereas $p_{\alpha }$ represents the code’s structural assignment (supply). This choice ensures that all amino acids used ($f_{\alpha }>0$) are represented within the code’s capacity ($p_{\alpha }>0$). While *D* could be simplified to cross-entropy (as the Shannon entropy of $f_{\alpha }$ remains constant), KLD is preferred here for its numerical interpretability, as it attains a meaningful minimum of zero when codon assignments perfectly align with proteomic demand.

#### Fitness function for the genetic code.

Finally, we define the fitness function *F* as a linear combination of error load *E* and amino acid KLD *D*:


$$\begin{array}{c} \begin{array}{cc} F & =-E-\eta D\\ & =-\sum_{i,j}\mu_{ij}|\phi_{i}-\phi_{j}{|}^{n}-\eta \sum_{\alpha \in AA}f_{\alpha }\ln\frac{f_{\alpha }}{p_{\alpha }}. \end{array} \end{array}$$ (5)

The parameter η serves as a balancing coefficient that modulates the relative contributions of these two selective forces. By tuning η, we can systematically assess whether internal error minimization (*E*) or external compositional demand (*D*) exerts a more dominant influence on the structural evolution and functional stability of the genetic code.

The linear, additive form of *F* is the most parsimonious scalarization of a two-objective problem: it posits a constant marginal trade-off between *E* and *D* and introduces only the single parameter η. Any nonlinear alternative (for example, $F=-E^{a}-\eta D^{b}$) would add free parameters whose biological interpretation is harder to justify, and we have no empirical basis for preferring a specific nonlinear form. Our central comparisons, moreover, do not hinge on this choice: the Pareto-optimal set of codes is fixed by the dominance relation among the (*E*,*D*) pairs and is therefore independent of the scalarization, so the position of the SGC relative to the attainable (*E*,*D*) frontier is unaffected by the linear form. The role of η is only to select where along that frontier the search is directed.

### Theoretical framework for local optimality

To evaluate the evolutionary stability of the SGC, we first examined its optimality within its immediate neighborhood. In our simulations, the per-codon misreading probability was fixed at *c* = 10^-4^ [72], and the mistranslation weights were normalized to maintain this overall probability even as the third-position transition weight ($w_{tt}$) varied. Given that *c* is small, the contribution of multiple-base misreadings to the translational error load is negligible compared to single-base misreadings. This analytical tractability allows us to approximate the critical threshold, $w_{tt}^{*}$, at which the SGC emerges as a local fitness peak. We conducted this analysis across absolute (*n* = 1), squared (*n* = 2), and generalized *n*-power error metrics.

The theoretical thresholds for $w_{tt}^{*}$ are derived as follows:

- Absolute error

$$\begin{array}{c} w_{tt}^{*}\approx \max\left(\sum_{i\in ℵ}w_{i}\right), \end{array}$$ (6)

- Squared error

$$\begin{array}{c} w_{tt}^{*}\approx \max\left(\frac{2}{\delta }\sum_{i\in ℵ}w_{i}{\tilde{\phi }}_{i}-\sum_{i\in ℵ}w_{i}\right), \end{array}$$ (7)

- *n*-power error

wtt*≈max{∑i∈ℵwi∑k=1n(−1)k−1(nk)|ϕ~iδ|n−k}. (8)

In these expressions, ${\tilde{\phi }}_{i}\equiv \phi_{i}-\phi_{\kappa }$, where $\phi_{i}$ denotes the polar requirement of the amino acid specified by codon *i*, and $\phi_{\kappa }$ is that of the amino acid originally specified by codon κ. The codon κ refers to the codon whose assigned amino acid changes as a result of local variation. The set ℵ represents the codons that are neighbors of κ, i.e., codons differing from κ by a single base substitution, excluding those involved in a third-position transition relationship and stop codons. δ denotes the difference in polar requirement between the original amino acid specified by codon κ and the substituted amino acid. In the SGC, the theoretical minimum of δ is 0.1 (e.g., reassigning a Cys codon to Leu), which serves as a critical scaling factor in higher-order error models (*n* > 1). Detailed mathematical derivations for these thresholds are provided in S1 Appendix.

### Optimization and simulation protocols

To characterize the global fitness landscape beyond the SGC’s local neighborhood, we implemented a stochastic search framework designed to evaluate code optimality across diverse evolutionary regimes. This approach involves a large-scale statistical assessment of the combinatorial code space and the identification of high-fitness architectures through global optimization.

#### Simulated annealing.

To move beyond local variation around the SGC and comprehensively characterize the fitness landscape of genetic codes, it is imperative to investigate the properties of its local optima. From an evolutionary perspective, natural selection typically favors trajectories that increase fitness, eventually driving the molecular code toward a stable local optimum. Consequently, characterizing the distribution of these optima provides critical insights into the evolutionary constraints that have shaped the SGC’s current architecture. However, exhaustively examining every possible optimum within such a complex topology is a formidable challenge. We therefore employ simulated annealing to sample high-fitness codes and characterize the global landscape structure efficiently.

Optimizing the genetic code is a high-dimensional combinatorial problem in which the number of potential configurations increases exponentially. Due to the ruggedness of the fitness landscape, the system exhibits numerous local optima that differ significantly in their proximity to the global peak. While some have fitness values comparable to the global optimum, others represent substantially lower fitness regions or metastable states [36,37]. To navigate this landscape efficiently, we adopt simulated annealing, a stochastic optimization technique that identifies high-quality solutions while avoiding poor local optima. By probabilistically accepting moves that temporarily decrease fitness, particularly during the high-temperature initial phase, simulated annealing effectively explores the global structure of the landscape. As the temperature gradually decreases according to a predefined schedule, the algorithm progressively converges toward stable, high-fitness architectures. This stochastic search mechanism is therefore expected to provide a more robust evaluation of the code’s error-minimizing properties compared to traditional deterministic methods.

**Algorithm 1 Simulated annealing**

1: **Inputs:**

Standard deviation of random code data: σ

Number of iterations: *n*

Initial temperature: $T_{i}=10\sigma$

Stop temperature: $T_{s}=10^{-4}\sigma$

Cooling rate: $\beta \leftarrow (T_{s}/T_{i}{)}^{1/n}$

2: **Initialize:**

$T\leftarrow T_{i}$

Generate a random initial code: $C_{R}$

3: **while** $T>T_{s}$ **do**

4:    Generate a random neighboring code of $C_{R}$: $C_{N}$

5:    Calculate fitness: $F(C_{R})$, $F(C_{N})$

6:    **if** $F(C_{N})\geq F(C_{R})$ **then**

7:     p←1

8:    **else**

9:     $p\leftarrow \exp\left[T^{-1}(F(C_{N})-F(C_{R}))\right]$

10:    **end if**

11:    With probability *p*, set $C_{R}\leftarrow C_{N}$

12:    T←β·T

13: **end while**

The optimization algorithm employed in this study follows the standard simulated annealing framework, with hyperparameters calibrated to ensure consistent optimization conditions across different η values. Specifically, we set the initial temperature $T_{i}$ proportional to the standard deviation of fitness values calculated from an ensemble of one million random codes, ensuring that the balance between exploration and exploitation remains consistent even as the fitness landscape dispersion varies with η. To maintain a fixed computational budget of *n* iterations across all runs, we design the annealing schedule with the cooling rate β determined as the *n*-th root of the temperature ratio $T_{s}/T_{i}$.

To place *E* and *D*, whose raw magnitudes differ by orders of magnitude, on a common footing, we standardized each as a *z*-score relative to the random-code ensemble, using the mean and standard deviation of 10^6^ random codes generated per species (see below). This standardization is a comparative device only; we do not assume that selection acts on *z*-scored objectives. The evidence that the SGC is non-random is established by its position within the random-code distribution. To characterize how the balance between error minimization and demand satisfaction shapes the resulting optimal codes, we ran the optimization at seven values of $\tilde{\eta }$ spanning six orders of magnitude (10^−3^ to 10^3^). Note that $\tilde{\eta }\equiv (\sigma_{D}/\sigma_{E})\eta$ is introduced to account for the scale difference between *E* and *D*, using the standard deviations $\sigma_{E}$ and $\sigma_{D}$ estimated from the ensemble of random codes.

#### Random code generation.

To evaluate the statistical significance of the SGC and its variants within the vast combinatorial space of possible mappings, we generated a null distribution of genetic codes. Unlike the deterministic optimization of the SGC, this randomization framework allows for an unbiased assessment of how the minimization of error load (*E*) and amino acid KLD (*D*) varies across different phylogenetic lineages and code types. Specifically, we calculated the distributions of *E* and *D* for each of the 28 species in our dataset by generating 10^6^ random codes per species. The random codes were constructed using a partitioning scheme that preserves the natural requirement of encoding 20 canonical amino acids:

1. Let $m=64-n_{\text{stop}}$ be the number of sense codons in the code being simulated (e.g., *m* = 61 for the SGC). These *m* codons are randomly permuted to remove any positional bias.
2. We uniformly choose 19 cut points from the m−1 available gaps between adjacent codons in the permuted list. This procedure partitions the *m* codons into 20 contiguous, non-empty blocks, effectively sampling an ordered composition of *m* into 20 positive parts from (m−119) possible configurations.
3. Each of the resulting 20 blocks is assigned to a specific amino acid in a fixed order, thereby defining a unique codon-to-amino acid mapping.

## Results

### The SGC is a local optimum under biased mistranslation

To determine whether the structural organization of the SGC is intrinsically adapted to the position- and transition-dependent bias of natural misreading, we first evaluated its stability within its immediate neighborhood. Analytical calculations using our derived framework reveal that for the absolute error metric (*n* = 1), the SGC emerges as a local fitness peak when the third-position transition weight exceeds $w_{tt}^{*}\approx 4.2$. This critical threshold is governed by the glutamic acid (Glu) codons (GAA/GAG), whose single-base neighbors carry the largest aggregate misreading weight. In sharp contrast, for the squared error metric (*n* = 2), the threshold rises roughly 50-fold to $w_{tt}^{*}\approx 214.1$, a value determined by the cysteine (Cys) codons (UGU/UGC), where the minimal polar requirement difference (δ=0.1) creates a bottleneck for local optimality.

**Fig 3:**
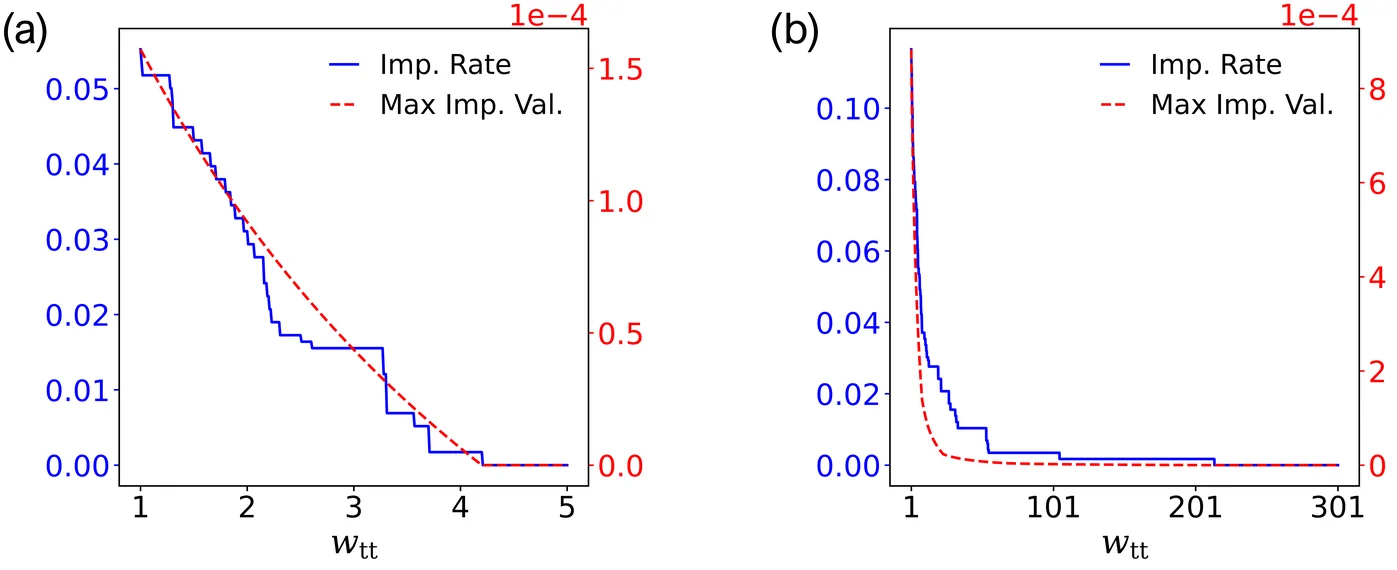
The error load of local variation around the SGC as the third-position transition weight ($w_{tt}$) increases. **(a)** Analysis using the absolute error (|Δϕ|) as the amino acid distance metric. **(b)** Analysis using the squared error ($|\Delta \phi {|}^{2}$) as the metric. In both panels, the solid blue line represents the improvement rate, defined as the fraction of the single-codon neighboring codes that exhibit a lower error load than the SGC. The dashed red line indicates the maximum improvement value, corresponding to the largest reduction in error load achievable among these neighbors for a given $w_{tt}$. Both metrics converge to zero beyond a threshold $w_{tt}^{*}$, demonstrating that the SGC achieves strict local optimality under high transition bias.

These theoretical predictions are in precise agreement with the numerical simulations presented in Fig 3, where the improvement rate vanishes exactly at the predicted $w_{tt}^{*}$ values. This scaling behavior can be mathematically attributed to the $\delta^{1-n}$ term in Eq 8; as δ reaches its minimum in the SGC, higher-order metrics (*n* > 1) exponentially inflate the transition bias required to sustain optimality.

This alignment between the theoretical threshold $w_{tt}^{*}\approx 4.2$ and the empirical range of transition bias (2≤γ≤3) provides a robust justification for employing the absolute error metric (*n* = 1) to model genetic code evolution. While higher-order metrics such as squared error (*n* = 2) require implausibly high mistranslation biases to sustain the SGC’s local optimality, the linear cost model yields a stability regime that is remarkably consistent with the actual magnitudes of decoding-error biases encountered in vivo. This indicates that the evolutionary constraints shaping the genetic code are best approximated by a fitness landscape where the penalty for amino acid substitutions scales linearly with their physicochemical differences.

**Fig 4:**
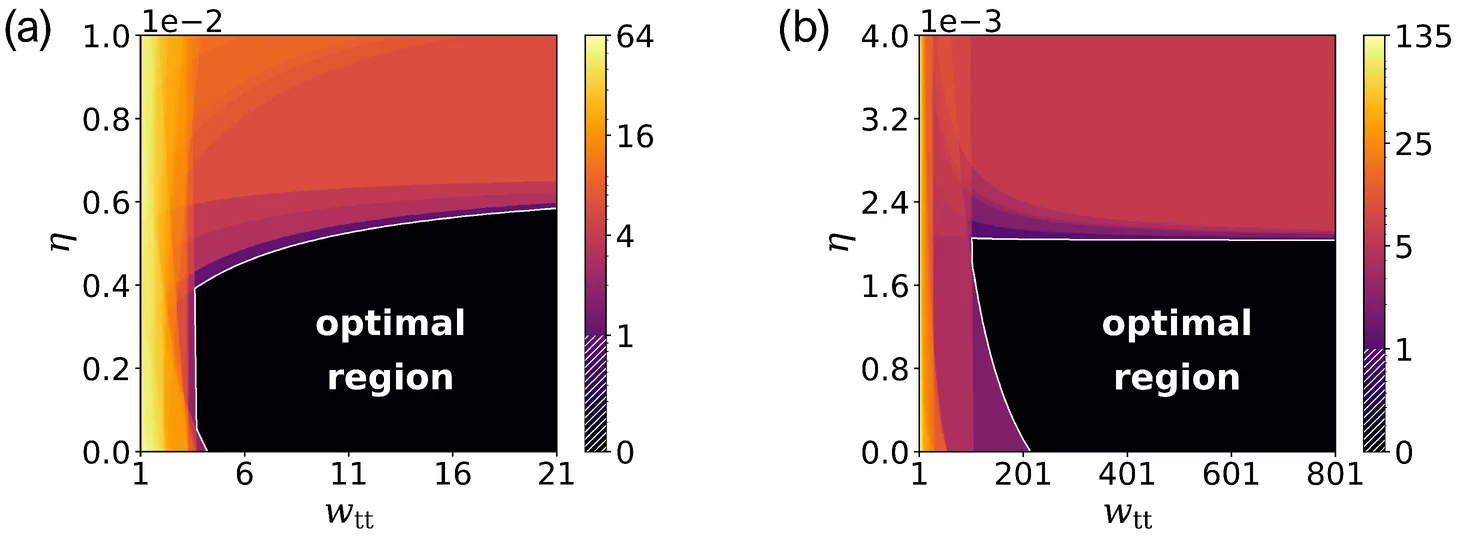
Landscape of local optimality for the SGC across the parameter space of the third-position transition weight ($w_{tt}$) and the balancing parameter (η). **(a)** Analysis using the absolute error (|Δϕ|) as the amino acid distance metric. **(b)** Analysis using the squared error ($|\Delta \phi {|}^{2}$) as the metric. The color bar represents the number of neighboring codes (out of 1,159 single-codon variants) with higher fitness than the SGC, displayed on a logarithmic scale. The black area, explicitly labeled “optimal region”, corresponds to the parameter regime where this count drops to zero, signifying that the SGC acts as a strict local optimum. Note that the color bar scale omits the interval (0, 1) to distinguish the optimal state from suboptimal states clearly.

To examine the combined effects of error load (*E*) and amino acid KLD (*D*), we extended the local variation analysis to the full fitness landscape by varying both $w_{tt}$ and the balancing parameter η (Fig 4). Each point in the heatmap represents the number of neighboring codes that outperform the SGC under the given parameter combination. For both absolute error (Fig 4a) and squared error (Fig 4b), the boundary of the SGC’s locally optimal region exhibits two characteristic features: a steep segment near $w_{tt}\approx w_{tt}^{*}$ and a nearly horizontal segment across a narrow η range. The steep boundary reflects the sharp increase in superior neighboring codes as $w_{tt}\to 1$, consistent with the relaxation of selective pressure for robustness against third-position transition errors. In contrast, the horizontal segment arises when $w_{tt}$ is sufficiently large that error load becomes dominated by third-position transitions, rendering fitness insensitive to further increases in transition bias.

These results yield two key insights into the genetic code’s optimization landscape:

First, absolute error (*n* = 1) applied to polar requirement differences $|\Delta \phi {|}^{n}$ sustains the SGC’s local optimality under biologically plausible mistranslation biases. While the analysis of $w_{tt}^{*}$ in the preceding section considered error load alone, the complete fitness landscape incorporating amino acid KLD confirms that absolute error maintains the SGC’s optimality at substantially lower $w_{tt}^{*}$ values than squared error, rendering the former metric more compatible with realistic transition-transversion biases.

Second, the SGC occupies a strict local optimum only when η is low, indicating that error minimization exerts stronger selective pressure than compositional matching. In particular, examining the regions where the SGC becomes a local optimum in the heatmaps of Fig 4 suggests that the selective pressure to minimize amino acid KLD operates independently of the SGC’s optimality. This decoupling is further corroborated by the simulated annealing experiments and code distribution analysis.

However, local variation analysis cannot fully capture evolutionary dynamics, as it assumes static parameters and incremental changes. To understand how the genetic code might have evolved, or could evolve under alternative selective regimes, we must characterize the global fitness landscape and its response to shifting environmental pressures. Accordingly, we next investigate the general characteristics of optimal codes and their dependence on key evolutionary parameters.

### Simulated annealing balances translation error and amino acid demand

The local variation analysis presented in Fig 4 reveals that the parameter regime where the SGC resides as a local optimum is characterized by a relatively low balancing parameter η. Notably, as the weight of amino acid demand (η) increases from zero, the local optimality of the SGC is preserved only through a concomitant increase in $w_{tt}^{*}$. This suggests that within the basin of attraction occupied by the SGC, selective pressure targeting only the minimization of compositional demand (*D*) is insufficient to fully account for its current structural properties.

Nevertheless, it is essential to recognize that the fitness function employed here serves as a first-order approximation of the multifaceted selective pressures that act throughout the evolutionary history of the genetic code. Consequently, the range of η values derived from this local variation framework should be interpreted as a comparative baseline rather than an absolute threshold. Rather than attempting to determine whether the SGC corresponds to a strict global optimum, we use this framework as a relative measure to assess the SGC’s proximity to the optimal frontier within the fitness landscape.

**Fig 5:**
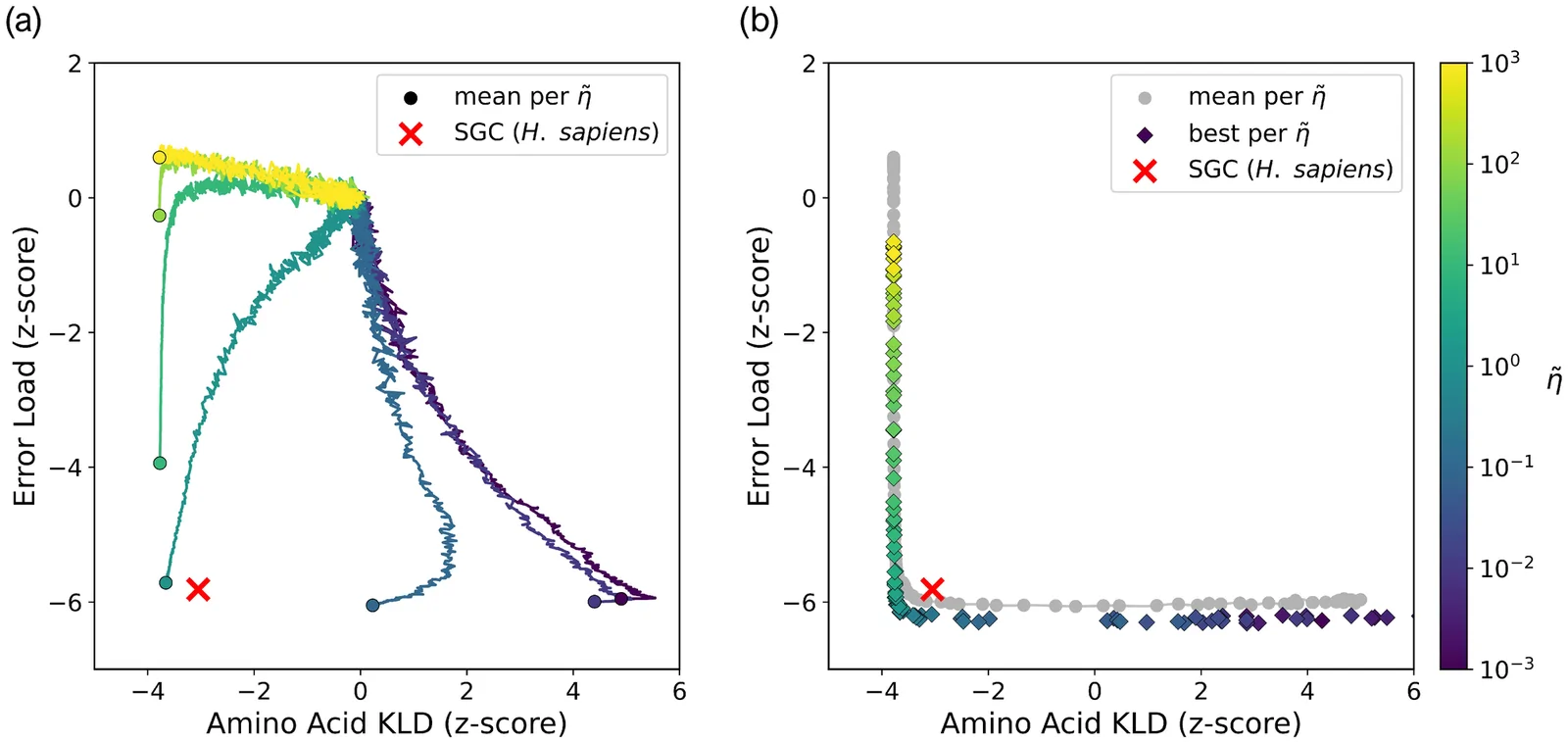
Optimization of the genetic code under varying balancing parameters. The normalized parameter $\tilde{\eta }\equiv (\sigma_{D}/\sigma_{E})\eta \approx 40.6\;\eta$ standardizes the two objectives by their random-code standard deviations; both axes are *z*-scores relative to the random-code ensemble, computed from *Homo sapiens* frequencies on 61 sense codons. The red cross marks the SGC, and color encodes $\tilde{\eta }$. **(a)** Mean simulated-annealing trajectories for seven values of $\tilde{\eta }$ spanning 10^−3^ to 10^3^, each averaged over 100 independent runs from random initial codes; the paths fan out from the random origin toward opposite corners of the attainable set as $\tilde{\eta }$ varies, and the filled circles mark each trajectory’s converged (final mean) position. **(b)** The near-continuous Pareto-like frontier traced by a dense $\tilde{\eta }$ sweep: the gray points and curve give the mean position per $\tilde{\eta }$, and the diamonds the best-fitness code per $\tilde{\eta }$. The SGC lies near the knee of the frontier, in the region reached at intermediate values of $\tilde{\eta }$.

The global optimization trajectories in Fig 5a display a clear dichotomy governed by $\tilde{\eta }$. In high-$\tilde{\eta }$ regimes the search minimizes *D*, driving it well below $D_{SGC}$, while the error load *E* is barely reduced and remains near its random-code mean ($z_{E}\approx 0$); in low-$\tilde{\eta }$ regimes the reverse holds, with *E* reaching values comparable to or below $E_{SGC}$ at the cost of a large increase in *D*. That sweeping the weight moves the optimum from one objective to the other is a generic feature of optimizing any monotone trade-off between two competing objectives, and we read it as confirmation that *E* and *D* are genuinely in tension under our objective rather than as a property peculiar to codon space. The asymmetry between the two corners, however, reflects the structure of the attainable (*E*,*D*) set: minimizing *E* without a demand constraint is most effectively achieved by maximizing redundancy, assigning most codons to a single amino acid, which collapses the amino acid vocabulary and sharply inflates *D*, whereas minimizing *D* merely matches the demand distribution and leaves *E* near its random-code level.

At intermediate $\tilde{\eta }$, the optimization is instead drawn to a region where *E* and *D* are both close to their minima, near the coordinates of the SGC. Fig 5b collects the optimal codes into a sharply L-shaped Pareto-like frontier: one arm reaches low *D* at high *E* (high $\tilde{\eta }$), and the other reaches low *E* at high *D* (low $\tilde{\eta }$). The SGC (red cross) lies near the knee joining these arms, well separated from either extreme, so it behaves as neither a pure error-minimizer nor a pure demand-satisfier and instead attains near-minimal values of both objectives at once. The existence and sharpness of this knee, and the SGC’s proximity to it, are properties of the empirical (*E*,*D*) landscape rather than artifacts of the additive fitness: the linear form neither creates the knee nor places any particular code near it.

### Genetic codes of species occupy exceptionally rare and optimal regions

**Fig 6:**
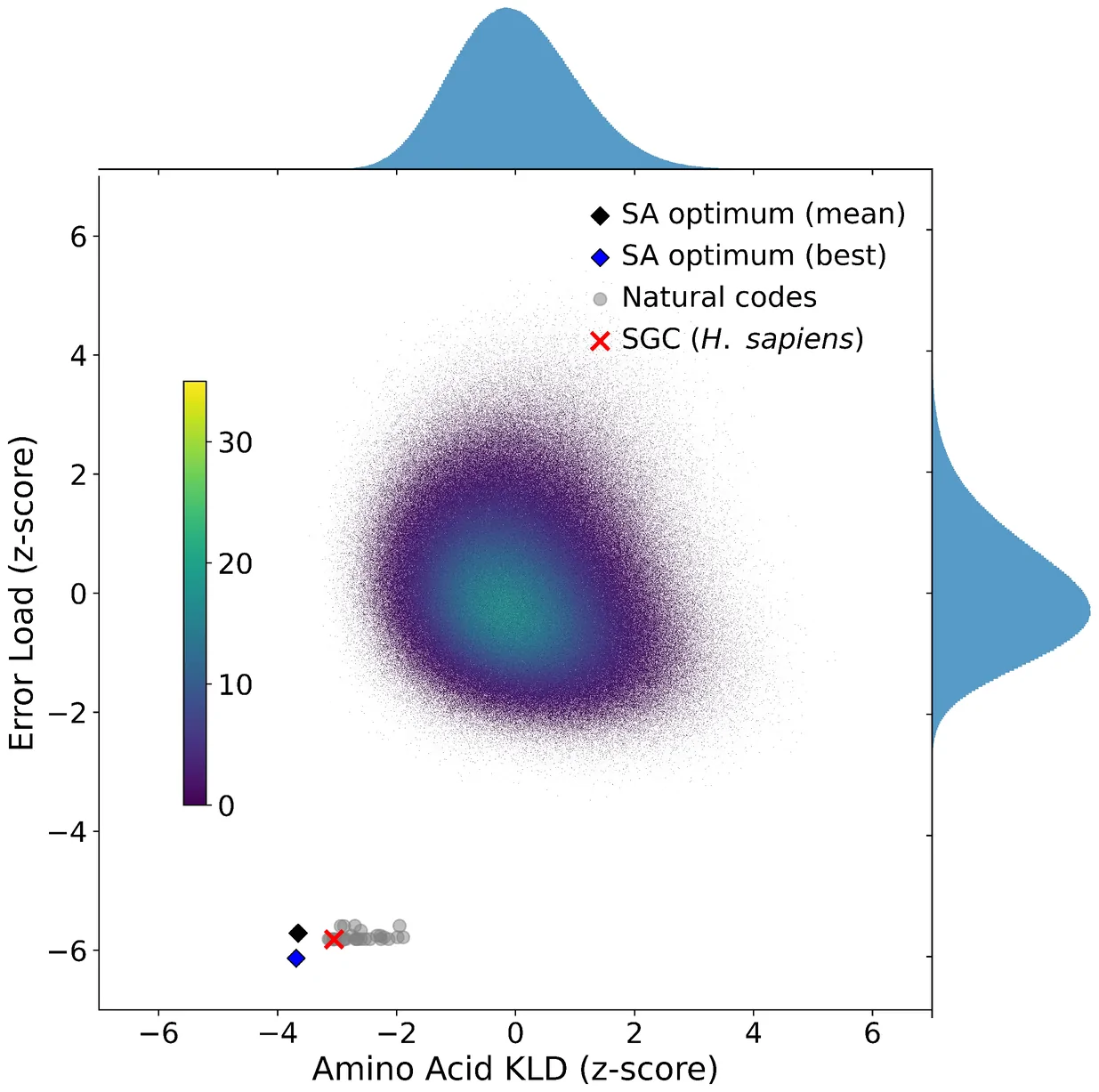
Joint distribution of error load (*E*) versus amino acid KLD (*D*) for genetic codes. The density heatmap shows the ensemble of $2.8\times 10^{7}$ randomly generated codes, constructed by partitioning *m* sense codons into 20 amino acid groups (10^6^ samples per species). Marginal histograms (top and right) illustrate that both objectives follow positively skewed distributions in the random ensemble. Gray circles denote the 28 natural codes (including variants), and the red cross marks the SGC (*H. sapiens*). For 1,000 optimal codes obtained via simulated annealing (at the balanced trade-off $\tilde{\eta }=1$), the black diamond marks their mean position and the blue diamond the single best-fitness code among them. Both metrics are presented as *z*-scores standardized against the random code distribution. The natural codes cluster tightly in the lower-left region, alongside the simulated-annealing optima, indicating that they are optimized for the simultaneous minimization of translational error and compositional mismatch.

To contextualize the evolutionary standing of natural genetic codes, we first analyzed the joint distribution of error load (*E*) and amino acid KLD (*D*) generated from $2.8\times 10^{7}$ random codes (Fig 6). The results reveal that these two objectives are nearly independent (Pearson’s r=−0.139), implying that the optimization of translational robustness is mechanically decoupled from the satisfaction of proteomic composition. Both metrics exhibit positively skewed, approximately skew-normal distributions due to distinct combinatorial constraints. Under our randomization scheme, the partitioning of codons typically yields a block-size distribution weighted toward small blocks, inflating *D* and creating a long right tail. Conversely, low values of *E* require globally coherent clustering of physicochemical properties across the codon-adjacency graph, which is a rare geometric arrangement that occupies a vanishingly small fraction of the code space. The observed skewness is empirically larger for *E* than for *D* (approximately 2.1 vs. 1.4 in our data). Remarkably, natural genetic codes (gray circles) cluster tightly in the lower-left region of this distribution, separated from the random bulk. This extreme positioning implies that the SGC did not emerge solely through neutral drift [73].

**Fig 7:**
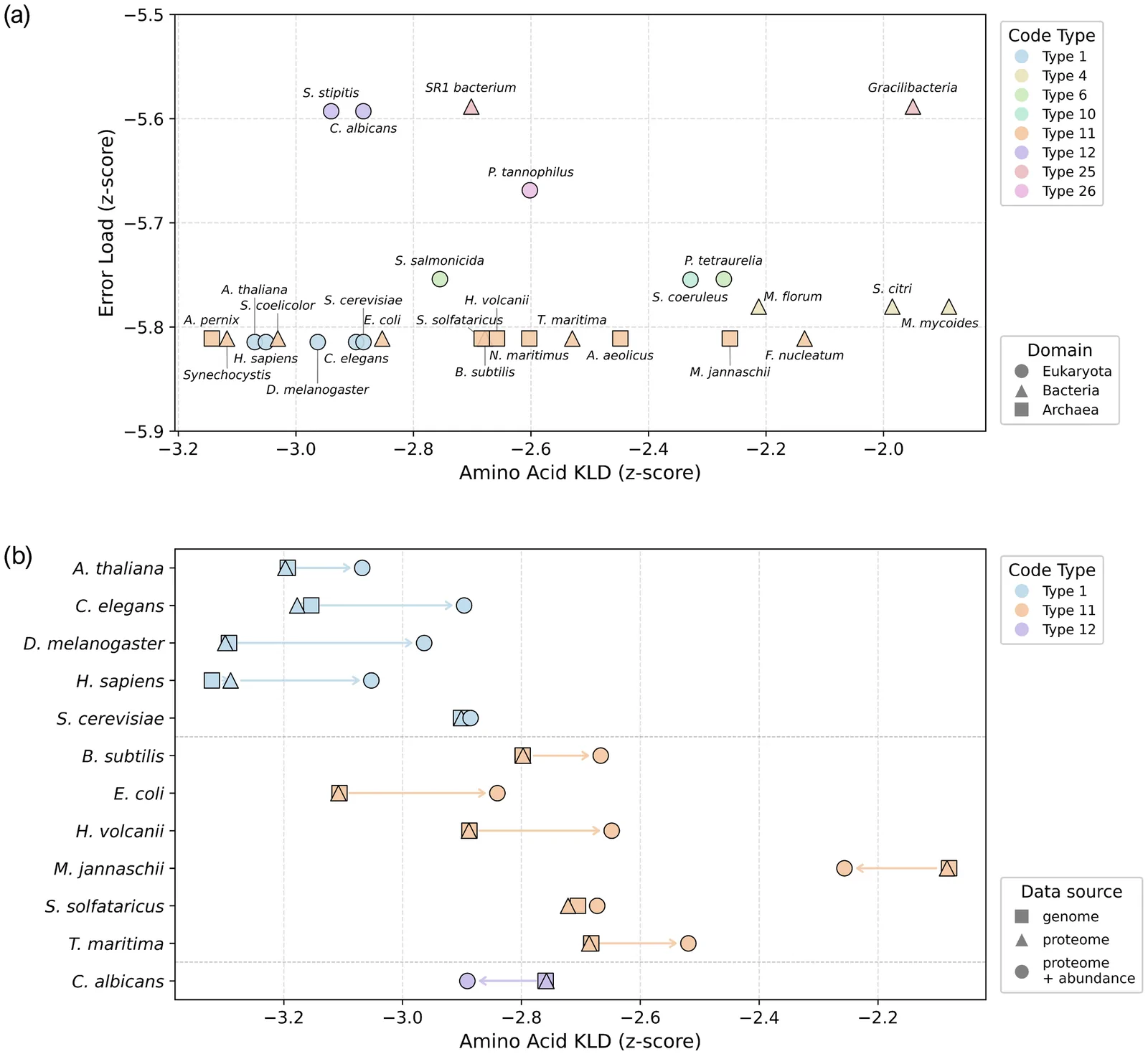
Per-species error load and amino acid demand of natural codes. **(a)** Standardized error load (*E*) versus amino acid KLD (*D*) for the 28 species, an enlarged view of the natural-code cluster in Fig 6. Marker color denotes the NCBI genetic code type and marker shape the domain (Eukaryota, Bacteria, Archaea); species are labeled by name (taxonomy IDs in S1 Table). *E* is fixed within each code type, forming horizontal bands, whereas *D* varies across species. **(b)** Robustness of *D* to the choice of demand data. For a representative subset of species spanning code types 1, 11, and 12, the amino acid KLD *z*-score is shown under three estimates of $f_{\alpha }$ (genome-derived CDS, UniProt reference proteome, and proteome weighted by protein abundance); arrows trace how *D* shifts across these estimates. The *D z*-scores remain strongly negative throughout, confirming that the demand-matching signal is robust to the data source.

Fig 7 displays standardized *z*-scores of error load (*E*) and amino acid KLD (*D*) for 28 species across eight genetic code types [45]. A primary regularity is that *E* remains constant within each code type, forming discrete horizontal bands that reflect the dependence of error robustness solely on codon-amino acid mapping, independent of species-specific amino acid usage. Among all surveyed codes, the SGC (type 1) and the bacterial/archaeal code (type 11), which share the same amino-acid assignments, attain the most negative *z*-scores for *E*, indicating the highest error robustness. The variant codes (types 4, 6, 10, 12, 25, 26) exhibit progressively less negative *z*-scores of *E*, implying that codon reassignments have generally incurred a modest but systematic cost in translational fidelity.

Within each code type, *D* varies across species, reflecting lineage-specific amino acid demands $f_{\alpha }$. Among standard-code (type 1) eukaryotes the values are tightly clustered (*Homo sapiens* −3.05, *Arabidopsis thaliana* −3.07, *Drosophila melanogaster* −2.96, *Caenorhabditis elegans* −2.90, *Saccharomyces cerevisiae* −2.89), indicating broadly conserved amino acid usage across these lineages. The bacterial and archaeal type-11 codes span a wider range, from strongly demand-matched genomes such as *Aeropyrum pernix* (−3.14), *Synechocystis* sp. (−3.12), and *Streptomyces coelicolor* (−3.03) to the more weakly matched *Fusobacterium nucleatum* (−2.13) and *Methanocaldococcus jannaschii* (−2.26). The least negative *D* values are found largely among the AT-rich Mollicutes (type 4: *Mycoplasma mycoides* −1.89, *Spiroplasma citri* −1.99, *Mesoplasma florum* −2.21) and in the reduced-genome candidate-division bacteria (type 25: *Candidatus* Gracilibacteria (Minisyncoccota) −1.95), whose skewed nucleotide composition yields the poorest match between $f_{\alpha }$ and the code-induced supply $p_{\alpha }$. Even so, the *D z*-scores of all 28 species remain clearly negative: every genome encodes amino acids far more in line with its proteome than a random code would. This indicates that *D* is shaped largely within codes, by lineage-specific composition, while intrinsic code optimization sets a strongly negative baseline shared across the tree of life.

Taken together, Fig 7 indicates a practical decoupling between code identity and lineage-specific proteome demand: code type discretely fixes *E*, while *D* varies within each code because it is driven primarily by differences in $f_{\alpha }$ rather than by the (nearly constant) $p_{\alpha }$. Species with low *D* are those whose amino acid usage is closer to the code-imposed baseline. At the same time, higher *D* reflects lineage-specific biases (e.g., nucleotide composition, metabolic or ecological constraints). Because $f_{\alpha }$ is estimated from heterogeneous data sources across species (abundance-weighted proteomes, unweighted proteomes, or coding sequences; S1 Table), cross-taxon comparisons of *D* should be made cautiously; the robustness of each species’ *D* to the data source (Fig 7b) nonetheless indicates that these choices do not materially affect the conclusions.

In this light, we interpret *D* as capturing compatibility between codon-imposed amino acid supply and organismal demand, complementing error robustness *E* in a multi-objective view of code evolution. The variant codes examined here do not display a consistent shift toward lower *D* relative to SGC-bearing lineages, lending no support to the hypothesis that recent reassignments were primarily selected to reduce *D*. Rather, reassignments appear to have been tolerated within constraints imposed by translational robustness and lineage-specific machinery, with clade-specific $f_{\alpha }$ potentially influencing which changes were permissible. Overall, Fig 7 is consistent with a scenario in which *E* imposes a strong code topological constraint, while *D* is shaped largely within codes as proteomes diverge across lineages.

## Discussion

This study examined the origin and organizing principles of the standard genetic code (SGC) through multi-objective optimization, balancing translational fidelity and amino acid diversity. The present analysis incorporates realistic mistranslation rates that vary by codon position and type, along with the amino acid composition observed in living organisms. We quantified translational error load (*E*) and compositional discrepancy (*D*) between observed amino acid usage and that implied by codon assignments, explicitly treating *D* as a key analytical variable.

Whereas Crick attributed the specific codon assignments to historical chance [5], subsequent work emphasizing translational error minimization [14,26,27] argued that they are instead shaped by selection. Our findings point to a further axis of organization: compositional matching between codon-imposed amino acid proportions and proteome usage. Local variation analysis (Fig 4) shows that the SGC reaches a local optimum for relatively small values of the balancing parameter η in the fitness function F=−E−ηD. The optimization using a simulated annealing algorithm (Fig 5) enables a more detailed analysis of the η value. Together, these results indicate that the SGC resides in a highly optimized region of the fitness landscape, balancing error minimization and compositional alignment. In this sense, codon degeneracy plays a dual role: it reduces mistranslation errors while matching codon multiplicity to amino acid usage frequencies, so that uniform codon usage naturally yields the empirical amino acid composition.

We interpret *E* as robustness to mistranslation rather than to DNA point mutation—a distinction worth making explicit, since the two processes differ in mechanism, in rate, and in the unit to which each rate refers. Replication errors accumulate per base per generation (roughly $\begin{array}{c} 10^{-9}-10^{-10} \end{array}$ in bacteria [74]), whereas misreading occurs per codon per translation event ($\sim 3\times 10^{-4}$ in *E. coli* [72] and $\sim 10^{-3}$ in eukaryotes [75]). These rates are not directly comparable; the relevant quantity is the number of erroneous protein molecules each process generates. A mutation alters a gene only once per replication, whereas every transcript is translated many times, so even a per-codon misreading rate of $\begin{array}{c} 10^{-3}-10^{-4} \end{array}$ makes mistranslation the overwhelmingly more frequent source of aberrant proteins at any moment. It is therefore mistranslation that imposes the dominant moment-to-moment pressure on the code’s error-minimizing topology, and the Freeland–Hurst weights we adopt were formulated for precisely this process. That point mutation shares the same qualitative transition and position biases is what makes the mutational γ a legitimate guide to the plausible weight range.

Our results provide a mechanistic explanation for how the genetic code maintains stability while allowing evolutionary adaptation. The joint distribution analysis (Fig 6) and the survey of natural variants (Fig 7) reveal a functional decoupling between *E* and *D*. The SGC is exceptionally rare with respect to both *E* and *D*, with the improbability of its error load particularly striking. From $2.8\times 10^{7}$ random codes, *E* and *D* are found to be negligibly correlated; although *D* is less statistically extreme than *E*, its *z*-scores remain negative. These results are consistent with the SGC’s organization having been shaped by selection that balances error load and compositional discrepancy. Specifically, we observed that *E* acts as a rigid global constraint determined by code topology, while *D* serves as a flexible variable that fluctuates with lineage-specific proteomic requirements.

This decoupling is biologically significant. It implies that the universal genetic code provides a robust scaffold (*E*) that ensures translational fidelity across all life forms, while offering sufficient compositional flexibility (*D*) to accommodate diverse proteomic strategies (e.g., GC-rich thermophiles vs. AT-rich intracellular parasites). Thus, beyond achieving low error load and demand mismatch, this flexibility may itself have contributed to the code’s persistence across lineages.

Several limitations constrain the interpretation of our model. First, the simulated annealing trajectory is a heuristic device for characterizing the fitness landscape on which the SGC resides, not a reconstruction of its evolutionary history; consistent with the frozen-accident view, we do not claim that the code continued to optimize after it froze, only that its present structure lies near an optimum of this landscape. Second, our framework isolates two selective forces, error minimization and compositional demand; coevolutionary and stereochemical mechanisms, together with codon-usage-based robustness analyses [40], remain complementary to rather than excluded by our account. The constant balancing parameter η and the simplified error model do not capture the full temporal complexity of billion-year evolution, and our analysis focused on the nuclear genetic code; extending it to mitochondrial and chloroplast systems could reveal distinct optimization landscapes shaped by their reduced genomes.

A further concern is the potential circularity between the supply $p_{\alpha }$ and the demand $f_{\alpha }$. Had $f_{\alpha }$ been tallied from coding sequences alone, it would share the codon composition that defines $p_{\alpha }$, so that part of their agreement could be arithmetic rather than evolutionary. To avoid this, we estimate $f_{\alpha }$ for most species from mass-spectrometry-based protein abundances (PaxDb), which are measured independently of the codon table and therefore decouple demand from supply. Reassuringly, the code’s compositional demand is essentially unchanged across the genome-, proteome-, and abundance-based estimates (Fig 7b). The lineage-specific variation in *D* also argues against a circular artifact: the *z*-scores of *D* are negative for all 28 species, yet vary substantially across lineages, which would not be expected if the match were a fixed consequence of codon arithmetic. Because the genetic code mediates all protein synthesis this circularity cannot be removed entirely, but these results indicate that it does not drive our conclusions. A related limitation is that all three of these estimates rest on either coding sequences or the steady-state proteome; because mistranslation acts per translation event, demand weighted by translational activity (transcript abundance and ribosome occupancy) would provide a more direct proxy for the fidelity pressure we invoke. Such translation-level data, however, remain concentrated in a few dozen, predominantly model, organisms [76,77] and are unavailable for most of the diverse lineages our panel is designed to compare, so incorporating them as they accumulate across taxa is left to future work.

In general, this work establishes a simplified landscape model for comparing possible code architectures under two objective functions: mistranslation robustness and amino-acid compositional compatibility. Our local variation analysis proposes that preserving synonymous codon blocks is critical for maintaining stability, a feature that allows the code to balance the rigidity needed for accurate translation with the flexibility required for proteomic diversity. By explicitly treating amino acid demand as a second optimization objective, we provide evidence that the standard genetic code is well described as a near-optimal solution to a multi-objective optimization problem. Future experimental validations using engineered translation systems, combined with more granular co-evolutionary simulations, are needed to further elucidate the dynamic interplay between the genetic code and the proteomes it encodes.

## Supporting information

S1 AppendixAnalytical derivation of the local optimality threshold.Derivation of the optimality threshold ($w_{tt}^{*}$) and Proof of Eqs 6–8.(PDF)

S2 AppendixRobustness of the error-minimization signal across amino-acid property scales.Joint distribution of error load (*E*) and amino acid KLD (*D*) for the random-code ensemble, the 28 natural codes, the SGC, and the simulated-annealing optima ($\tilde{\eta }=1$) under four physicochemical property scales (polar requirement, hydropathy, molecular volume, and isoelectric point), with accompanying description and analysis. The mean error-load *z*-score of the natural codes is −5.77 (PR), −7.06 (hydropathy), −5.53 (volume), and −2.59 (isoelectric point), confirming that the error-minimization signal is robust to the choice of amino acid property.(PDF)

S1 Table Species dataset. Taxonomic classification, genetic code type, data source, and sequence statistics for the 28 species analyzed in the code distribution analysis.
(CSV)
