# Supplementary material for: The genetic code at the balance point of error and demand
Source: PLoS Comput Biol. 2026 Aug 11;22(8):e1014613. doi: 10.1371/journal.pcbi.1014613 (PMC13497292; doi:10.1371/journal.pcbi.1014613)
Supplement: Supplementary file 1 [file pcbi.1014613.s001.pdf]

S1 Analytical derivation of the local optimality threshold

Since all codons are robust to third-position transition misreadings, any local variation that alters the encoded amino acid necessarily disrupts this robustness. Assuming that double or higher-order misreadings are negligible ($c^2 \ll c$), the error load is affected only by the amino acid distance between the varied codon and its neighboring codons that differ by a single-base misreading. Let κ represent the codon altered by the local variation. Since each of the three codon positions allows one transition and two transversion misreadings, κ has up to nine neighboring codons. This number may decrease to eight or seven if any of the neighboring codons correspond to stop codons. Let $\aleph$ denote the set of neighboring codons, excluding those that arise from third-position transition misreadings or correspond to stop codons. Let w_i represent the misreading weight associated with each codon i in this set.

In the case of absolute error, the amino acid distance is determined by the difference in polar requirement values ϕ between the amino acids specified by two codons. Let the polar requirement of the amino acid specified by codon κ change from ϕ_κ to ϕ_κ^* due to local variation. Then, the corresponding change in error load, ΔE, can be approximated as follows:

$$\begin{aligned}\Delta E\left(\phi_\kappa \to \phi_\kappa^*\right) &\approx \sum_i w_i\big(|\phi_i - \phi_\kappa^*| - |\phi_i - \phi_\kappa|\big) \\ &= \sum_{i\in\aleph} w_i\big(|\phi_i - \phi_\kappa^*| - |\phi_i - \phi_\kappa|\big) + w_{\mathrm{tt}}\big(|\phi_{\mathrm{tt}} - \phi_\kappa^*| - |\phi_{\mathrm{tt}} - \phi_\kappa|\big).\end{aligned} \tag{S1}$$

Let $\delta \equiv \phi_\kappa^* - \phi_\kappa$ and $\tilde{\phi}_i \equiv \phi_i - \phi_\kappa$. Then, ΔE becomes:

$$\Delta E\left(\phi_\kappa \to \phi_\kappa^*\right) \approx \sum_{i\in\aleph} w_i\left(\left|\tilde{\phi}_i - \delta\right| - \left|\tilde{\phi}_i\right|\right) + w_{\mathrm{tt}}\left(\left|\tilde{\phi}_{\mathrm{tt}} - \delta\right| - \left|\tilde{\phi}_{\mathrm{tt}}\right|\right). \tag{S2}$$

For the SGC to qualify as a local optimum, it must satisfy $\Delta E \geq 0$ for all κ. Since robustness to third transition misreadings is established across all codons, we have $\tilde{\phi}_{\mathrm{tt}} = 0$. Given that w_{tt} is the largest among the six weights, the threshold value w_{tt}^* at which the SGC becomes a local optimum is determined as follows:

$$w_{\mathrm{tt}}^* \approx \max\left\{\frac{1}{|\delta|}\sum_{i\in\aleph} w_i\left(\left|\tilde{\phi}_i\right| - \left|\tilde{\phi}_i - \delta\right|\right)\right\}. \tag{S3}$$

From Eq. (S3), a necessary condition for the expression inside the braces on the right-hand side to be maximal is that, for all i, either $\phi_i \leq \phi_\kappa^* < \phi_\kappa$ or $\phi_i \geq \phi_\kappa^* > \phi_\kappa$. This implies that ϕ_κ must be either the maximum or minimum among all ϕ_i, and that ϕ_κ^* is the second-largest or second-smallest, respectively. Hence, under this condition, we have $\left|\tilde{\phi}_i\right| - \left|\tilde{\phi}_i - \delta\right| = |\delta|$, and thus

$$w_{\mathrm{tt}}^* \approx \max\left\{\sum_{i\in\aleph} w_i\right\}. \tag{S4}$$

Analyzing the structure of the SGC reveals that the codons UGU and UGC, which encode cysteine (Cys, $\phi = 4.3$) and exhibit the lowest polar requirement, are connected via a third position transversion misreading ($w_3^{\mathrm{tv}} = 1$) to the neighboring stop codon UGA. In contrast, the codons GAA and GAG, which encode glutamic acid (Glu, $\phi = 13.6$) and exhibit the highest polar requirement, are connected via a first position transversion misreading ($w_1^{\mathrm{tv}} = 0.5$) to the neighboring stop codons UAA and UAG.

Accordingly, the threshold value is estimated as $w_{\mathrm{tt}}^{*} \approx 4.2$ under the assumption of local variation in GAA or GAG.

The value of w_{tt}^{*} can also be derived analogously in the case of squared error. The change in error load, ΔE, takes the same form as that in Eq. (S1) for absolute error, except that the absolute difference in polar requirement is replaced by the square of the difference:

$$\begin{aligned}\Delta E\left(\phi_{\kappa} \to \phi_{\kappa}^{*}\right) &\approx \sum_{i} w_i \left[\left(\phi_i - \phi_{\kappa}^{*}\right)^2 - \left(\phi_i - \phi_{\kappa}\right)^2\right] \\ &= \sum_{i \in \aleph} w_i \left[\left(\tilde{\phi}_i - \delta\right)^2 - \left(\tilde{\phi}_i\right)^2\right] + w_{\mathrm{tt}} \left[\left(\tilde{\phi}_{\mathrm{tt}} - \delta\right)^2 - \left(\tilde{\phi}_{\mathrm{tt}}\right)^2\right] \\ &= \sum_{i \in \aleph} w_i \delta\left(\delta - 2\tilde{\phi}_i\right) + w_{\mathrm{tt}} \delta\left(\delta - 2\tilde{\phi}_{\mathrm{tt}}\right)\end{aligned} \tag{S5}$$

Similarly, since $\tilde{\phi}_{\mathrm{tt}} = 0$, the threshold value w_{tt}^{*} is given by:

$$\begin{aligned}w_{\mathrm{tt}}^{*} &\approx \max\left\{\frac{1}{\delta^2} \sum_{i \in \aleph} w_i \delta\left(2\tilde{\phi}_i - \delta\right)\right\} \\ &= \max\left\{\frac{2}{\delta} \sum_{i \in \aleph} w_i \tilde{\phi}_i - \sum_{i \in \aleph} w_i\right\}.\end{aligned} \tag{S6}$$

The expression inside the brackets is maximized when the signs of δ and $\tilde{\phi}_i$ are identical for all i, $|\delta|$ is minimized, and $\left|\tilde{\phi}_i\right|$ is maximized. Given the values of polar requirement, the minimum possible value of $|\delta|$ is 0.1. To maximize $\left|\tilde{\phi}_i\right|$, ϕ_{κ} must be either the maximum or minimum among all polar requirement values. In the SGC, this condition is satisfied by the codons UGU and UGC, which specify the amino acid with the lowest polar requirement ($\phi = 4.3$) and yield $|\delta| = 0.1$. Accordingly, under the assumption of local variation in these codons, the resulting threshold value is calculated as $w_{\mathrm{tt}}^{*} \approx 214.1$.

Having analyzed w_{tt}^{*} under absolute and squared error, we now generalize the formulation to the case where the amino acid distance is defined as $|\Delta\phi|^n$. As in the previous two cases, we assume that the codons specifying the amino acid with the maximal or minimal ϕ_{κ} undergo local variation such that ϕ_{κ}^{*} becomes the second-largest or second-smallest value. Under this assumption, and applying the binomial theorem, the change in error load ΔE can be expressed as:

$$\begin{aligned}\Delta E\left(\phi_{\kappa} \to \phi_{\kappa}^{*}\right) &\approx \sum_{i} w_i \sum_{k=1}^{n} (-1)^k \binom{n}{k} \left|\tilde{\phi}_i\right|^{n-k} |\delta|^k \\ &= \sum_{i \in \aleph} w_i \sum_{k=1}^{n} (-1)^k \binom{n}{k} \left|\tilde{\phi}_i\right|^{n-k} |\delta|^k + w_{\mathrm{tt}} |\delta|^n.\end{aligned} \tag{S7}$$

Setting $\Delta E = 0$, the threshold value w_{tt}^{*} is given by:

$$w_{\mathrm{tt}}^{*} \approx \max\left\{\sum_{i \in \aleph} w_i \sum_{k=1}^{n} (-1)^{k-1} \binom{n}{k} \left|\frac{\tilde{\phi}_i}{\delta}\right|^{n-k}\right\}. \tag{S8}$$

Assuming that $|\delta| \ll \left|\tilde{\phi}_i\right|$, the threshold value w_{tt}^{*} can be approximated by retaining only the leading-

order term of the binomial expansion:

$$w_{\mathrm{tt}}^* \sim \max\left\{ n \sum_{i \in \aleph} w_i \left| \frac{\tilde{\phi}_i}{\delta} \right|^{n-1} \right\}. \tag{S9}$$

Since the minimum possible value of $|\delta|$ is 0.1, the threshold value w_{tt}^* increases rapidly as the exponent n increases. Moreover, UGU and UGC are the only codons that satisfy $|\delta| = 0.1$ and either maximize or minimize $\left|\tilde{\phi}_i\right|$. Therefore, for $n \geq 2$, the codons that determine the threshold value for the third-position transition weight are uniquely fixed as UGU and UGC.
