# Supplementary material for: The genetic code at the balance point of error and demand
Source: PLoS Comput Biol. 2026 Aug 11;22(8):e1014613. doi: 10.1371/journal.pcbi.1014613 (PMC13497292; doi:10.1371/journal.pcbi.1014613)
Supplement: Supplementary file 2 [file pcbi.1014613.s002.pdf]

S2 Robustness of the error-minimization signal across amino-acid property scales

The error load E used throughout this work is defined on the Woese polar requirement (PR), the physicochemical property on which genetic-code error minimization was originally quantified (Woese et al., 1966; Haig and Hurst, 1991) and which underlies the influential "one in a million" analysis of Freeland and Hurst (1998). Polar requirement is, however, only one of several physicochemical scales commonly used to assess the mutational and translational robustness of the genetic code. To verify that our conclusions are not specific to this choice, we repeated the random-code distribution analysis under the four properties highlighted in the distortion analysis of Radványi and Kun (2021): polar requirement, hydropathy (Kyte and Doolittle, 1982), molecular volume (Grantham, 1974), and isoelectric point (pI). For each scale we recomputed the error load of every random and natural code and re-standardized it against the corresponding random-code ensemble. The amino acid KLD term D depends only on the proteomic demand and the codon partition, not on the property scale, and is therefore identical to the main analysis.

S2 Fig shows that the natural codes occupy the extreme low-error tail of the random-code distribution under every scale. The mean error-load z-score of the 28 natural codes is -5.77 under polar requirement, -7.06 under hydropathy, -5.53 under molecular volume, and -2.59 under isoelectric point. The error-minimization signal is therefore strong and consistent across polar requirement, hydropathy, and molecular volume, in fact strongest under hydropathy, and, although weaker under isoelectric point, it still places the natural codes far below the random expectation. This pattern is expected on prior grounds: the genetic code is organized principally around amino acid polarity and hydrophobicity, the axis captured by polar requirement and hydropathy and the property for which code error minimization was first demonstrated (Woese et al., 1966; Haig and Hurst, 1991; Freeland and Hurst, 1998). Properties less aligned with this axis, such as isoelectric point, consequently yield a weaker — though still clearly significant — signal, which is fully consistent with the established structure of the code.

The simulated-annealing optima recovered under each property (black and blue diamonds) converge on the same low-(E,D) region occupied by the SGC and the remaining natural codes. The SGC therefore lies close to the attainable optimum under every property examined, not only under polar requirement. Because D is scale-independent, the horizontal positions of the natural codes and of the SGC are identical in all four panels; the variation across panels is confined to the error-load axis and reflects only how each property maps onto the codon-adjacency structure of the code.

Taken together, these results demonstrate that the error-minimization signal central to our conclusions is robust to the choice of amino acid property: the natural codes are pronounced low-error outliers, and the SGC sits near the optimal frontier, regardless of the specific physicochemical scale. We retain polar requirement as the single measure reported in the main text, both for its canonical status and to avoid a redundant battery of strongly correlated measures in the principal analysis, rather than because it is uniquely favorable.

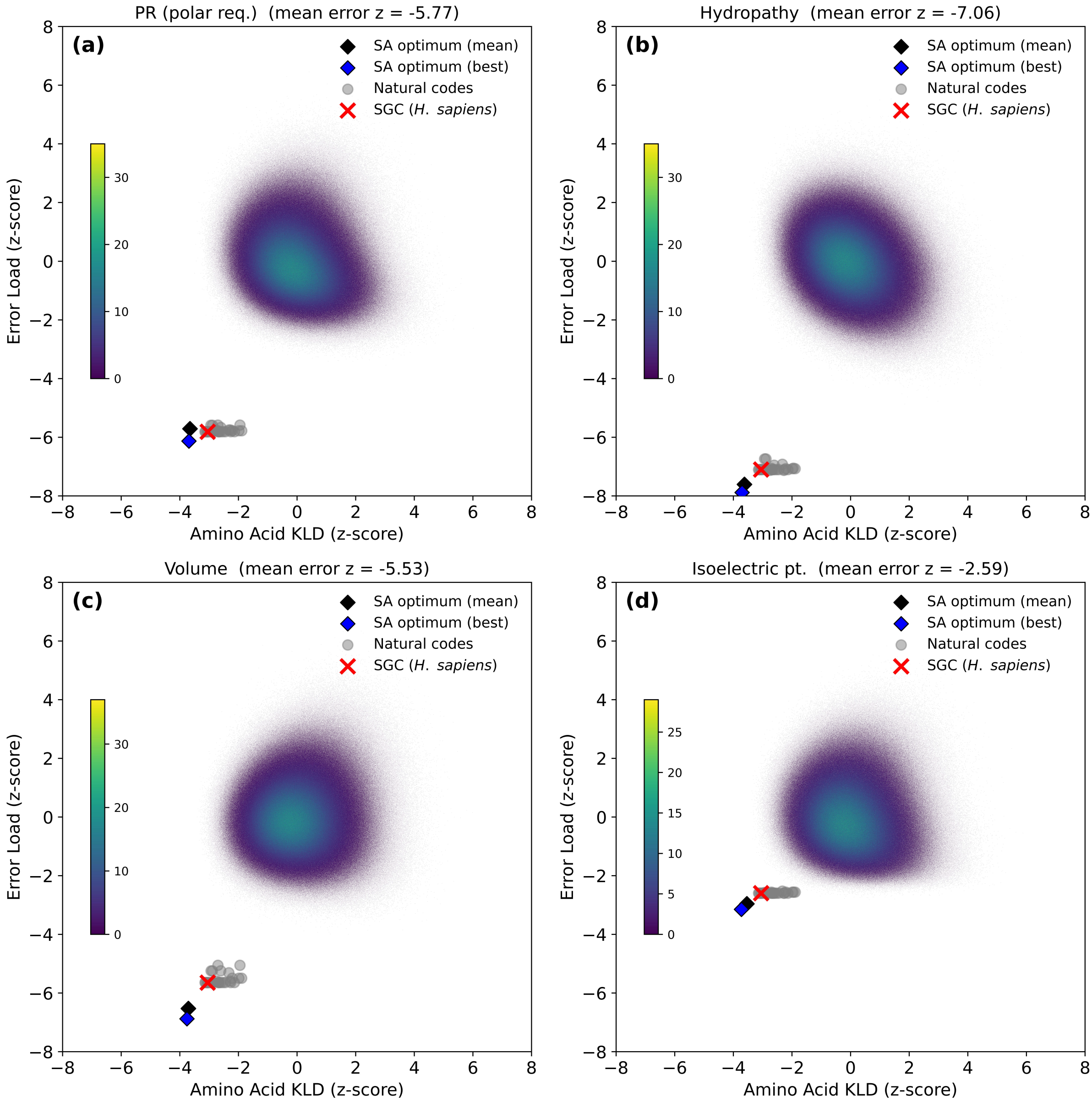

S2 Fig. Joint distribution of error load and amino acid KLD under four amino-acid property scales. Each panel shows, for one physicochemical property, (a) polar requirement (PR), (b) hydropathy (Kyte–Doolittle), (c) molecular volume (Grantham), and (d) isoelectric point (pI), the random-code ensemble in the standardized (D, E) plane (z-scores relative to the random ensemble, as in **Fig 6** of the main text). The viridis density heat map (inset color bar: number of random codes per bin) gives the joint distribution of amino acid KLD (D, abscissa) and error load (E, ordinate); gray circles mark the 28 natural codes; the red cross marks the SGC (*H. sapiens*); the black and blue diamonds mark, respectively, the mean and the single best-fitness genetic code obtained by simulated annealing under that scale at the balanced trade-off $\tilde{\eta} = 1$ (1,000 independent runs per scale). Because D depends only on the amino acid demand and the codon partition, it is identical across panels; only the error-load axis changes with the property. Panel titles report the mean error-load z-score of the 28 natural codes: -5.77 (PR), -7.06 (hydropathy), -5.53 (volume), and -2.59 (pI).
