# Supplementary material for: The genetic code at the balance point of error and demand
Source: PLoS Comput Biol. 2026 Aug 11;22(8):e1014613. doi: 10.1371/journal.pcbi.1014613 (PMC13497292; doi:10.1371/journal.pcbi.1014613)
Supplement: Supplementary file 3 [file pcbi.1014613.s005.pdf]

Response to the Reviews and Decision

Title: The genetic code at the balance point of error and demand (originally submitted as: "Balancing translational fidelity and amino acid demand in the evolution of the genetic code")

Manuscript Reference Number:
PCOMPBIOL-D-26-00560

Authors:
Yudam Seo
Tsvi Tlusty
Junghyo Jo

Date: June 15, 2026

Message from the Authors

Dear Editors and Reviewers,

We appreciate the constructive feedback that has helped us improve the quality of our manuscript. We have addressed these comments and incorporated your valuable suggestions into the revised manuscript. In the revised manuscript, updates can be tracked using latexdiff to distinguish them from the original text.

In the detailed responses below, we address each comment individually. Comments received are indicated in boxes, and our responses are provided immediately following each comment. Unless otherwise noted, all line numbers and reference numbers in the responses are based on the revised manuscript. Line numbers and reference numbers cited in the reviewers' comments remain unchanged and are based on the original manuscript. We look forward to your feedback and hope you find the revised manuscript satisfactory.

Sincerely,
Yudam Seo, Tsvi Tlusty and Junghyo Jo.

Response To Editor

Overall Comments

> Thank you for submitting your manuscript to PLOS Computational Biology. After careful consideration, we feel that it has merit but does not fully meet PLOS Computational Biology's publication criteria as it currently stands. Therefore, we invite you to submit a revised version of the manuscript that addresses the points raised during the review process. It is especially important that you clarify the questions related to the fitness function, and the relation of your results to prior work.

Response

We sincerely thank you for the positive evaluation of our manuscript and for the opportunity to submit a revised version. We have carefully revised and refined the manuscript with particular attention to the two key issues you highlighted: the fitness function and the relation of our work to prior studies.

Regarding the fitness function, two important concerns were raised: the circularity of D and the justification for defining the fitness function as a linear combination. With respect to the circularity of D, we have extended our analysis beyond the original genome-only framework by incorporating proteome and protein abundance data. While the involvement of the genetic code in the central dogma is fundamental, and circularity therefore cannot be entirely avoided, the introduction of protein abundance allows f_α to better reflect its intrinsic biological meaning, thereby substantially mitigating the circularity of D.

As for the definition of the fitness function, the central aim of this study is to identify codes that are jointly optimized with respect to two terms: the error load and the amino acid KLD. We now use the linear fitness only as a transparent scalarization of a two-objective problem; the main conclusion rests on the SGC's position in the (E, D) landscape, not on a privileged value of η. The resulting trade-off is clearly captured by the Pareto front, and Figure 5, which illustrates this relationship, has been refined accordingly.

Regarding the relation to prior work, we have also worked to clarify how our study connects with and builds upon existing research, as you suggested. First, in response to Reviewer 1's concern regarding terminological misuse, we have unified our description to frame the relevant quantity as a weighting for mistranslation—consistent with the original intent of Freeland & Hurst (1998) [29]—and have also corrected the scaling of the error probabilities. Second, we have reframed our treatment of the frozen accident theory: whereas our original framing emphasized the random, accidental nature of the code's fixation, we now emphasize that, once frozen, the code admitted only rare and minor changes—predominantly stop-codon reassignments that leave existing proteins essentially unchanged. Relatedly, drawing on studies of the environmental conditions under which the code is thought to have frozen, we have revised our species selection accordingly.

In addition to these major revisions, a number of more detailed changes have been made throughout the manuscript, which are described in further detail in our point-by-point responses to the reviewers' comments. We are once again grateful for the opportunity to revise our work, which has enabled us to submit a substantially improved manuscript.

Response To Reviewer #1

Reviewer Comment

> The paper under review deals with an exciting and yet contentious issue of the genetic code optimization level and the exact nature of evolutionary forces that shaped the code's table. The main contributions of the paper are 1) taking account the existing frequencies of the amino acids to emphasize a multi-criteria nature of the code optimization; while in some of the previous studies this metric was already present (to the best of my knowledge, the amino acid frequencies first appeared in Ref. [34], it seems that this fact should be highlighted more in the text), in the current text the empirical proteomic demands are taken into account; 2) the mutation weights (Table 2) used in this study are finally given somewhat more careful treatment compared to previous analysis (but also see below); 3) a novel fitness function (Eq. 5 in the text) is suggested to consider the code evolution as multi-criteria optimization under two evolutionary forces: to minimize the cost of the possible errors and to guarantee that the code table has the sufficient capacity to produce the necessary amino acids in required amounts.
>
> While I find the progress of the paper somewhat incremental I also think that the paper is carefully written, does a good job explaining the details of simulations, brings some relatively new ideas to the fields, and concludes with a non-surprising, and yet well defended conclusion that "[the standard genetic code] is a highly effective solution that balances fidelity against resource availability constraints." Additionally, the paper fits well the scope of the journal, and therefore I recommend accepting the paper for publication. I do not have any major comments about the chosen approaches simply because almost all of them can be questioned and defended at the same time.

Response

We would like to express our deepest gratitude to the reviewer for their meticulous review, positive evaluation of this paper, and recommendation for publication. You accurately identified the three key contributions of our research (multi-criteria optimization, application of mutation weights, and introduction of a new fitness function), and also endorsed the validity of our simulation process and final conclusions.

Furthermore, we fully agree with the reviewer's suggestion that the importance of Gilis *et al.* (2001) [39] regarding the consideration of amino acid frequencies should be addressed more clearly in the text. In tracing the historical development of this idea, we noted that the correlation between codon degeneracy and amino acid frequency was in fact first reported by King and Jukes (1969) [38], and that Gilis *et al.* (2001) themselves cite King and Jukes (1969) as the originators of this observation while describing their own work as an extension of it. To represent this lineage faithfully—and to ensure that readers clearly recognize that amino acid frequency has been a key parameter in previous studies of code optimality—we have added the following sentence to the Introduction (immediately preceding the Radványi and Kun (2021) [40] reference):

> The idea that amino acid frequencies themselves carry information about code optimality has a longer history: King and Jukes (1969) first noted the correlation between codon

degeneracy and the frequency of each amino acid in proteins, and Gilis *et al.* (2001) later formalized it by weighting amino acid substitution costs by empirical amino acid frequencies in a fitness function for code optimality, thereby establishing amino acid frequency as a quantitative parameter in such analyses.

Beyond the specific points above, we are also deeply thankful for your broad understanding of the academic limitations and complexities inherent in the research methodologies we adopted, as well as for your recognition of the overall value of this paper. All detailed comments provided have been carefully reviewed and incorporated into the manuscript.

Reviewer Comment

> My only major remark is about the authors' usage of the terms "mutations" and "mistranslations." For instance, the term "transition" means exactly a point mutation of a certain kind and does not refer to the possible mistranslation at the same position during the translation process. The mutation weights chosen by Freeland and Hurst (1998) [29] refer specifically to the mutation probabilities, not to mistranslations. My understanding is that the authors tacitly assume that the frequencies of mutations are the same as the frequencies of mistranslations and use these terms interchangeably. Such a bold generalization requires at least some discussion and references to the known data on both mutation and mistranslation rates. I think that the paper will benefit from such a discussion.

Response

We sincerely thank the reviewer for his/her careful review, which pointed out the inaccurate use of the terms "mutation" and "mistranslation" in the manuscript. We fully agree that treating these two biologically distinct processes as equivalent was an oversimplification.

In particular, we apologize for causing confusion by labeling the weights from Freeland and Hurst (1998) as "mutation weights" in Tables 1 and 2. As the reviewer astutely pointed out, point mutations (at the DNA/RNA level) and translation errors (i.e., errors arising during decoding by the translational process) arise from completely different biological mechanisms and exhibit a vast difference in their occurrence rates.

However, we would like to respectfully clarify a detail regarding the Freeland and Hurst (1998) model to justify our use of their parameters. As stated in their original paper (specifically in the section "Mapping Translational Error Data to MS Calculations"), the weights we adopted from their Table 2 were indeed formulated specifically to model mistranslation probabilities, based on in vitro translation data by Friedman and Weinstein (1964).

We note that Freeland and Hurst (1998) explicitly used the term "transition" in describing translational misreading bias. In their model, the final mistranslation weight is the product of two independent factors, the relative frequency and the transition weighting, which together approximate the conditional probability of a mistranslation event. The relative frequency reflects the position-dependent stringency of decoding (misreadings being rare at the second codon position and more frequent at the wobble third position), whereas the transition weighting reflects the greater propensity for transition over transversion misreadings.

To address your important points and ensure complete clarity, we have substantially revised the manuscript as follows:

Clarification of Terminology. We have revised the text throughout the Methods and Results sections. We now explicitly state that the weights used in our simulations (adopted from Freeland and Hurst, 1998) do not represent basal DNA point mutation probabilities, but rather mistranslation weights (translational error probabilities). In parallel, the misreading probability parameter in our reader matrix has been recalibrated to a translation-relevant order of magnitude: $p_{\text{mistrans}} = 10^{-4}$ per codon per translation event, instead of the previous value 10^{-10} (which was more consistent with DNA point-mutation rates). All downstream quantities (error load E, the range of η, and the σ_D/σ_E scaling factor) have been recomputed accordingly. The z-score-based conclusions, including the SGC's exceptional position in the random-code distribution, are invariant under this rescaling, so the qualitative conclusions of the original submission are preserved while the parameter values now reflect a biologically meaningful interpretation.

Addition of the Requested Discussion. Following the reviewer's constructive concern, we have added a new paragraph to the Discussion section (Lines 600–614) that explicitly contrasts the mechanisms and empirical rates of DNA point mutations versus translational errors. The new paragraph cites the following data:

- Replication errors accumulate per base per generation (roughly 10^{-9}–10^{-10} in bacteria (Drake *et al.*, 1998 [74])).
- Misreading occurs per codon per translation event ($\sim 3 \times 10^{-4}$ in *E. coli* (Kramer and Farabaugh, 2007 [72]) and $\sim 10^{-3}$ in eukaryotes (Drummond and Wilke, 2009 [75])).

Although these rates refer to different units and are not directly comparable, the fact that each transcript is translated many times means mistranslation generates far more abnormal protein molecules per unit time, which is why we treat it as the dominant pressure on the code's error-minimizing topology.

By clearly distinguishing between these two evolutionary error types and refining our model accordingly, we believe the manuscript now provides a more rigorous and biologically faithful treatment of code optimality.

Response To Reviewer #2

Reviewer Comment

> This manuscript presents a sophisticated computational analysis of genetic code optimality using multi-objective framework. While the core methodology is reasonable and the findings are generally expected, the biological interpretation of the balancing parameter η and the circularity inherent in defining amino acid demand from coding sequences require additional scrutiny and validation. The authors should address the circularity concern by validating D with independent data sources, provide stronger biological justification for the fitness function form, and clarify the distinction between optimization algorithms and historical evolutionary processes.

Response

We are deeply grateful to the reviewer for the thoughtful and rigorous critique of our manuscript. The four central concerns raised, (i) the potential circularity in defining the compositional demand D from coding sequences, (ii) the biological justification of the linear additive fitness form $F = -E - \eta D$, (iii) the calibration of η through z-score normalization, and (iv) the relationship between simulated annealing as a numerical algorithm and the actual evolutionary history of the SGC, target the methodological foundations of our analysis. We have substantially revised the manuscript in response to each of these points; the detailed individual responses follow below.

The circularity inherent in deriving f_α from CDS-derived sequences is the most consequential of these concerns, and also ties in with Reviewer #3's point. We address it by introducing an independent data source: protein abundance measurements from the PaxDb integrated dataset (Wang *et al.*, 2015 [46]). For 13 of our 28 species for which integrated proteomic abundance data are available, we now compute f_α as the abundance-weighted amino acid frequency across the entire UniProt reference proteome (for *Mesoplasma florum*, NCBI's `fasta_cds_aa` output is used since PaxDb maps via locus tags to that source). For the remaining 15 species, we fall back to the UniProt reference proteome (unweighted) or, if unavailable, to the NCBI CDS-translated frequency as in the original submission. The data source per species is recorded in a new "Source" column of Supplementary Table S1, allowing the reader to stratify the results by data quality.

This abundance weighting f_α is measured at the protein level through mass spectrometry–based quantification (rather than relying on NCBI genome data), and is therefore structurally decoupled from the supply p_α induced by the codon table. That said, even already-expressed proteins cannot be fully exempt from the latent influence of the genetic code; because this is an inherent feature of the protein synthesis machinery described by the central dogma, it is a problem that cannot be entirely avoided. Under this premise, our additional experiments show that the resulting D values and their z-scores relative to the random-code baseline do not deviate substantially from the unweighted estimates (new Fig. 7b), demonstrating that our key conclusions are robust to this refined validation.

Regarding the form of the fitness function, we address this point in detail in our response to a subsequent comment. We have also added a clarifying paragraph to the Discussion (Lines 632–637) that distinguishes between two things: the use of simulated annealing as a numerical tool for characterizing the fitness landscape of possible codes, and any direct claim about the temporal

trajectory of the actual evolutionary history of the SGC.

Reviewer Comment

> My main question is whether the arbitrary fitness definition affect the patterns observed. Although weighted by the eta parameter, it is still arbitrary to assume a linear additive contribution to F for D and E.
>
> [...]
>
> The results in 442-448 (also 461-465) seems to be a mathematical consequence of the Fitness being the linear additive combination of D and E, not necessarily related to the property of codon code space.

Response

We appreciate the reviewer's astute observation that the linear additive form of the fitness function, $F = -E - \eta D$, is one specific choice among many possible scalarizations of a two-objective optimization. We fully agree that this assumption deserves explicit justification, which we have added (Methods, Lines 314–323). Our defense rests on four observations:

(i) Linear scalarization is the canonical first-order treatment of multi-objective optimization. It assumes a constant marginal trade-off between the two objectives, which is the most parsimonious starting point. Any nonlinear extension (e.g., $F = -E^a - \eta D^b$ or $F = -\log E - \eta \log D$) would introduce additional free parameters whose biological interpretation is even harder to defend than that of a single η. In the absence of an empirical basis for a specific nonlinear form, the linear additive model serves as a transparent baseline.

(ii) The Pareto-optimal set is defined entirely by the dominance relation among the (E, D) vectors and is therefore independent of any choice of scalarization. Maximizing any strictly monotone aggregation $g(E, D)$ returns a point on this same frontier; only the location along the frontier depends on the form of g (with a linear g recovering its convex-supported portion). Consequently, the qualitative claim that the SGC lies near the empirical (E, D) Pareto frontier does not depend on our linear choice of F.

(iii) Lines 442–448 describe the optimization trajectories. We concede that the qualitative dichotomy is a generic consequence of optimizing any monotone trade-off between two competing objectives. In high-η runs the optimizer drives D down while E improves little, and in low-η runs the reverse occurs; sweeping the weight necessarily moves the optimum along the Pareto front from one anchor toward the other. This behavior is an algorithmic property of the scalarization, and we have reworded the passage to present it as a confirmation that E and D are genuinely in tension under our objective, rather than as a finding about codon space.

(iv) Lines 461–465 make a logically distinct, empirical claim about where the natural code falls within the attainable (E_z, D_z) set. The crucial point here is geometric rather than parametric, and it does not depend on the form of F. The empirical front is sharply L-shaped: one arm extends to high E at near-minimal D (high η), and the other extends to high D at near-minimal E (low η). The SGC lies near the knee that joins these two arms, well separated from either extreme. It therefore behaves as neither a pure error-minimizer nor a pure KLD-minimizer, and it instead attains near-minimal values of both objectives simultaneously. The existence and sharpness of this knee are properties of the empirical codon-amino-acid landscape, not features imposed by the

additive form: linearity neither creates a knee where the attainable set has none, nor places any particular code near it.

Accordingly, we have revised Lines 492–514 to separate the two statements explicitly. The trajectory dichotomy (492–500) is now presented as an algorithmic property of the scalarization, whereas the position of the SGC (508–514) is presented as a property of the empirical landscape. In the latter passage we have also replaced the earlier wording, which framed the SGC as a compromise in which demand restricts error minimization, with a description of the SGC as occupying a balanced position near the knee of the front, since this is what the data in Fig 5 actually show.

Reviewer Comment

> The fitness function $F = -E - \eta D$ employs a crucial parameter η that modulates the relative weight of error load versus compositional demand. The authors calibrate η using standardized z-scores (0.56) based on the assumption that the SGC represents a balanced state between selective pressures. This approach risks circular reasoning, assuming the SGC is optimal to define the parameter that then "proves" it is optimal. Moreover, there is no independent biological justification for why natural selection would operate on z-score-normalized objectives rather than raw fitness costs.

Response

We agree that calibrating η from SGC-derived z-scores risks circular reasoning, and in this revision we have removed the SGC-derived weighting from the manuscript entirely: no parameter is fixed from the SGC's own position. This dissolves the concern at its source. Importantly, doing so leaves our central conclusion untouched, because that conclusion never depended on η — the evidence that the SGC is non-random derives entirely from its position relative to the random-code baseline, independently of any choice of η. The role of $\tilde{\eta}$ is now confined to a sensitivity analysis in which we sweep it across six orders of magnitude, rather than fixing it at any SGC-calibrated value, and we ask only at which weightings the resulting optima resemble the SGC. We have revised the relevant sections (Methods, Lines 388–395; Results, Lines 486–491) accordingly. Our defense rests on three points:

(i) The optimality claim for the SGC does not depend on η. The empirical observation grounding our central conclusion, namely that the SGC occupies an exceptionally rare region of the (E, D) landscape, is established by comparing the SGC's (E, D) coordinates with the unconditional distribution of E and D over 2.8×10^7 random codes (28 species $\times$ 10^6 random codes per species). The resulting z-scores of the SGC ($z_E \approx -5.8$, $z_D \approx -3.1$) characterize its rarity before any η value is invoked. The parameter η enters only later, in our analysis of which direction of selection (the relative weighting of E vs D) best explains the SGC's position within the frontier of possible codes.

(ii) The alignment with the SGC emerges from the data, not from an assumed weight. Rather than calibrating a single $\tilde{\eta}$ from the SGC's coordinates, we sweep $\tilde{\eta}$ across seven values spanning six orders of magnitude (Fig 5a) and ask where the resulting optima fall. The SA optima approach the SGC's position only in the intermediate-$\tilde{\eta}$ regime; extreme weightings instead converge on qualitatively different corners of the attainable set (the high-redundancy corner at low $\tilde{\eta}$, the high-diversity corner at high $\tilde{\eta}$). Because each SA run starts from a random code and evolves under a fitness landscape defined entirely by the random-code ensemble, the proximity of the intermediate-$\tilde{\eta}$ optima to the SGC is a prediction validated against an external benchmark, not a tautology:

were the SGC typical of random codes, no value of $\tilde{\eta}$ would place the optimum near it. This $\tilde{\eta}$-dependence is thus an empirical pattern that cannot be reduced to circular reasoning.

(iii) z-score normalization is a comparative tool, not a biological mechanism. We do not claim that natural selection operates on z-score-normalized objectives. z-scores are used purely to render E and D comparable, given that their raw scales differ by orders of magnitude ($E \sim 10^{-3}$, $D \sim 10^{-1}$ under our mistranslation model). The set of Pareto-optimal codes is invariant under any positive affine rescaling of the individual objectives, such as z-scoring, because such transformations preserve the coordinatewise ordering on which dominance is defined; only the shape of the frontier in the plotted plane is rescaled, not which codes lie on it. Our qualitative conclusions are therefore unchanged whether one uses raw values or standardized scores. We have added explicit clarification of this point in the Methods (Lines 387–392).

Reviewer Comment

> Line 471. It is reasoned that the optimization of E without constraining D will lead to extremely low D with only one type of amino acid. However, D and E are statistically independent among random codes. Is this logically contradictory?

Response

We thank the reviewer for catching this contradiction, which warrants careful clarification. The reviewer correctly notes that E and D are statistically near-independent across the unconstrained random-code ensemble (Pearson's $r = -0.139$, line 470–473 of the original manuscript). At the same time, we argue that low-η optimization, which effectively minimizes E alone, drives the code toward configurations that concentrate codons into a small number of amino acids, thereby inflating D. The reviewer asks how both statements can be true simultaneously.

The apparent tension dissolves once one distinguishes marginal statistics from conditional behavior in the tails of the distribution. The Pearson correlation $r = -0.139$ summarizes the bulk of the random-code distribution; it does not characterize the tails, where simulated annealing under strong selective pressure necessarily operates. Our defense rests on two points:

(i) Marginal independence does not preclude tail anti-correlation. A random partition of 61 codons into 20 groups produces (E, D) values whose joint distribution has near-zero linear correlation overall. However, the geometric configurations that minimize E, namely those that concentrate codons into a few large groups corresponding to physicochemically similar amino acids, necessarily produce highly skewed group sizes, and hence large D. The lowest-E tail of the random-code distribution is therefore strongly enriched for high D, even though the bulk of the distribution shows no such correlation.

(ii) Optimization seeks the corner, not the centroid. Simulated annealing under low $\tilde{\eta}$ aggressively pursues low-E codes regardless of their typical D. Because the low-E tail is the high-D corner of the joint distribution (Fig. 6), the optimization trajectory necessarily ends in this corner. This corner-seeking behavior is fully consistent with the marginal near-independence of E and D across the bulk of random codes, rather than contradicted by it. We further note that this tail coupling is directional, in agreement with Fig. 5: minimizing E forces the amino acid distribution to concentrate and thus inflates D, whereas minimizing D merely matches the demand distribution and leaves E near its random-code mean ($z_E \approx 0$ at high $\tilde{\eta}$).

Reviewer Comment

> Line 82. The proteomic demand was derived from the codon usage frequency, or amino acid usage frequency?

Response

We thank the reviewer for highlighting an ambiguity in our original phrasing. To clarify, the proteomic demand D is derived from the amino acid usage frequency, not from the codon usage frequency. Specifically, f_α denotes the observed frequency of amino acid α in the (translated) proteome, and p_α denotes the codon-implied supply $|C_\alpha|/61$, where $|C_\alpha|$ is the number of sense codons assigned to amino acid α under the genetic code in question. The KLD is then $D = \sum_\alpha f_\alpha \log(f_\alpha/p_\alpha)$.

We also recognize that the reviewer's question reflects a genuine imprecision in the original submission. There, f_α was obtained by translating NCBI coding sequences (CDS) and tallying amino acid occurrences. Although this quantity is an amino acid frequency rather than a codon frequency, it was derived entirely from genomic coding sequences, in which each protein-coding gene contributes in proportion to its length but irrespective of its expression level, so that the abundances of the actually synthesized proteins play no role. Describing such a quantity as "proteomic demand" was therefore not fully accurate, and the reviewer's question is well founded.

In the revised manuscript we have introduced proteome and protein-abundance data, so that f_α now measures amino acid usage at the protein level in a genuine sense. It is obtained from one of three data sources, in order of preference:

1. UniProt reference proteome, weighted by per-protein abundance (PaxDb integrated dataset);
2. UniProt reference proteome, unweighted (when abundance data are unavailable);
3. NCBI CDS translations, as in the original submission (fallback when no curated proteome is available).

The data source for each species is recorded in the new "Source" column of Supplementary Table S1.

The first option, used for 13 of our 28 species, addresses the circularity concern most directly, since f_α is then derived from protein-level measurements that are mechanistically decoupled from the codon-to-amino-acid mapping. We elaborate on this point and its bearing on the circularity concern in our responses to the summary comment and to the reviewer's first comment.

Response To Reviewer #3

Reviewer Comment

> The authors rightfully list some of the theories governing the evolution of the genetic code. A full review of them could be found, for example, in here: `10.1016/j.biosystems.2017.10.006`. The two most important theories for the study at hand are the error minimization theory and the frozen accident theory. The importance of the first is obvious, but the second is also of importance. The authors wrote "This scenario suggests that the code's evolution arrested, or "froze," to preserve the functional integrity of protein synthesis". The importance of the frozen accident theory is this, and not random assignment of codons and amino acids. Once there are many peptides and the cell relies on them (keep in mind that the genetic code evolved in an RNA world, so peptides gradually took over functions from ribozymes), changes to the genetic code is very detrimental. Experiments to change the genetic code proves that while it can be done, there is a considerable fitness cost to it. Consequently, I think (and I'm not the only one) that further optimization once it "froze" was not possible. The slight tweaking seen in alternative genetic codes mostly affects the STOP/Termination codons (thus do not disturb the amino acid composition of existing peptides). I would include a brief overview of the alternative genetic code in the introduction.

Response

We are sincerely grateful to the reviewer for this thoughtful clarification of the frozen accident theory and for directing us to the comprehensive survey of Kun and Radványi (2018) [13], which assesses the competing hypotheses of code evolution and argues that the standard code is best understood as an integration of several mechanisms, notably a combination of the frozen accident with selection for translational error minimization. We fully agree that our original framing did not give sufficient weight to the frozen accident as a constraint on further evolution, as opposed to the random assignment of codons to amino acids. The reviewer's formulation is precisely the one we intended to invoke, and our original phrasing inadvertently conflated the two.

We have revised the Introduction (Lines 12–30) to:

1. Distinguish more clearly between the two questions traditionally addressed in genetic code evolution: why a single code is preserved (the frozen accident proper) versus how the specific codon-to-amino-acid assignments first arose (the question of initial conditions);

2. Acknowledge the experimental evidence, from synthetic genome-recoding and codon-reassignment studies, that altering the SGC is achievable but carries a considerable fitness cost, supporting the view that substantial further optimization after the freeze was effectively precluded;

3. Add a brief overview of the known alternative genetic codes, noting that the documented variants predominantly involve reassignment of stop codons (for example, UGA → Trp in *Mycoplasma*, NCBI table 4; UAG and UAA → Gln in ciliates, table 6; UGA → Cys in *Euplotes*, table 10), which leave the internal amino acid composition of existing proteins essentially unchanged. We present the rare sense-codon reassignments, such as CUG → Ser

in the *Candida* CTG clade (table 12), as the notable exceptions: these do alter amino acid composition and are thought to have been tolerated only through a gradual, ambiguous-decoding intermediate, consistent with the high fitness cost of code change emphasized by the reviewer;

We have also taken care to position our contribution within this integrated picture. Our analysis quantifies the error-minimization and compositional-demand components of code organization; we do not claim these to be the sole determinants, and we now state explicitly that the coevolutionary and stereochemical contributions reviewed by Kun and Radványi (2018) are complementary to, rather than excluded by, our results.

Reviewer Comment

> If we accept that at certain early point in the history of life on Earth, the SGC froze, then organisms a few billion years removed are poor proxy to study if the code is optimal. We can only use what we have, but at least we can try to find genomes that could be closer in composition to the one in which the SDG froze. One can posit that the SDG is rather optimal and find an environment in which it is so. It turns out that the SDG froze in a temperate environment (`10.3390/life11080773`). Consequently, the choice of species in the analysis is rather strange. While the authors claim phylogenetic diversity, they also wanted to pick the different alternate genetic codes. A quick glance at the list of alternative tables shows that most comes from ciliates (Eukaryotes, Alveolata). An interesting clade in the Eukaryotic tree, but what it has to do with the origin of the genetic code is very questionable (understatement). Instead of using a bunch of Eukaryotes and some strange bacteria, I would suggest employing a good mix of bacteria and archaea. Recent phylogenies of bacteria (`10.1126/science.abe0511`) and archaea (`10.1038/s41564-021-00918-8`) could help in the picking.

Response

We agree with the reviewer that the original species selection was poorly balanced and that the over-representation of ciliates is not a strong basis for inferring properties of the SGC's evolutionary origin. We have substantially revised our species panel in response to this criticism, guided in part by the references provided.

Revision of the species panel. The dataset has been pruned from the original 58 species to a curated set of 28 species, with the explicit goal of (i) reducing redundant ciliate and alveolate entries, (ii) increasing the representation of bacteria and archaea, and (iii) covering the phylogenetic breadth that supports the cited prokaryotic phylogenies (Coleman *et al.*, 2021 [42] for bacteria; Rinke *et al.*, 2021 [43] for archaea). The revised panel is composed as follows:

Domain	Species	Notes
Bacteria	12	spanning Firmicutes, Proteobacteria, Cyanobacteria, Actinobacteria, Mollicutes, Aquificae, Thermotogae, Fusobacteria, candidate division SR1
Archaea	5	spanning Crenarchaeota (*Aeropyrum*, *Saccharolobus*), Euryarchaeota (*Haloferax*, *Methanocaldococcus*), Thaumarchaeota (*Nitrosopumilus*)
Eukaryota	11	retained to anchor the comparison and provide non-standard genetic code variants (tables 6, 10, 12, 26) that have no known bacterial/archaeal counterpart

Treatment of alternative codes. Eukaryotic species are retained where strictly necessary to instantiate non-standard NCBI genetic codes (tables 6, 10, 12, 26) that are biologically confined to specific eukaryotic clades. In contrast, the bacterial/archaeal NCBI table 11, which shares the same codon-amino-acid mapping with table 1 (with differences only in start-codon recognition), is now represented by 12 of the 28 species, ensuring that the dominant clade of the tree of life is no longer underrepresented.

Mesophilic anchoring. Following Radványi and Kun (2021) [44], who argue that the SGC froze in a temperate environment, we have ensured that mesophilic genomes form a clear plurality of the bacterial/archaeal contingent, while still retaining a small number of thermophiles and halophiles to test the robustness of our conclusions to environmental extremes.

Robustness of conclusions. We emphasize that the error load E is a function only of the codon-to-amino-acid mapping and is therefore identical for all species sharing the same codon table; the species panel affects only the compositional demand D. The qualitative results — the SGC's exceptional position in the joint (E, D) distribution and its proximity to the Pareto frontier at intermediate $\tilde{\eta}$ — are preserved under the revised panel, as documented in the updated Figs. 6 and 7 and Supplementary Table S1.

Reviewer Comment

> Polar requirements is just one of the physicochemical properties usually used in assessing the mutational robustness of the genetic code. Why use this and not a mix of the usual ones, like hydropathy, molecular volume and isoelectric point (plus polar requirement)?

Response

We thank the reviewer for this question, which is entirely reasonable. We adopted polar requirement as the primary measure because it is the canonical property in this literature: it underlies the foundational quantifications of code error minimization (Woese *et al.*, 1966 [14]; Haig and Hurst, 1991 [28]) and the influential "one in a million" analysis of Freeland and Hurst (1998) [29]. Using it places our results in direct continuity with, and makes them directly comparable to, the body of work that established the error-minimization hypothesis.

To verify that our conclusions are not specific to this choice, we repeated the analysis under all four properties highlighted by the reviewer, namely polar requirement, hydropathy, molecular volume, and isoelectric point (the same set employed in the distortion analysis of Radványi and Kun, 2021 [40]). The results are presented in a new supplementary figure S2, reproduced here for the reviewer's convenience. In the joint (D, E) distribution over the random-code ensemble for all 28 natural codes (S2 Appendix), the natural codes fall far below the random-code mean in error

load under polar requirement, hydropathy, and molecular volume, with mean error-load z-scores of -5.77, -7.06, and -5.53, respectively. The error-minimization signal is therefore strong and consistent across these three measures.

Under isoelectric point the signal is weaker (mean error-load $z = -2.59$), although it still places the natural codes well below the random expectation. This is expected on prior grounds: the genetic code is organized principally around amino acid polarity and hydrophobicity, the axis captured by polar requirement and the property for which code error minimization was first quantified (Woese *et al.*, 1966; Haig and Hurst, 1991; Freeland and Hurst, 1998). Properties less aligned with this axis, such as isoelectric point, therefore yield a weaker, though still significant, signal. This is consistent with the established structure of the code and does not affect our conclusion.

Because the central result holds across these measures, the specific choice of property does not materially change our findings. We have therefore retained polar requirement as the single measure reported in the main text, both for its canonical status and to avoid a redundant battery of strongly correlated measures in the principal analysis, rather than because it is uniquely favorable. To make this robustness explicit and permanently available to readers, we have added the full four-property analysis as S2 Appendix, and we now refer to it in the main text (Lines 239–245).

Reviewer Comment

> Furthermore, there are studies of mutational robustness that consider the composition of proteomes (such as `10.1038/s41598-021-90440-y`). The authors should have a look at it.

Response

We are grateful to the reviewer for pointing us to Radványi and Kun (2021) [40], which we had not adequately considered. In that work the authors assess the mutational robustness of the genetic code using a distortion metric across amino acid physicochemical properties (polar requirement, hydropathy, volume, and isoelectric point), weighted by organism-specific codon usage. This is closely aligned with our premise that organism-specific composition modulates the selective pressure on the code, and it motivated us to incorporate proteome-level data and to verify that the compositional-demand component of our analysis remains robust under this richer characterization of amino acid usage.

In this revision we replaced the genome-derived (NCBI CDS) amino acid frequencies used in the original submission with proteome-level frequencies weighted by protein abundance (PaxDb integrated dataset) wherever available (13 of 28 species; see our response to the summary comment). Because the compositional demand D is computed directly from these frequencies, a natural concern is whether the SGC's demand-matching property, namely that its amino acid KLD lies far below that of the random-code ensemble, survives this change of data source, or whether it was an artifact of the original genome-based frequencies.

To address this directly, we have added a new panel, Fig 7b. Because the error load E is determined by the genetic code itself, it is unaffected by the choice of proteomic data; the component of our analysis that genuinely depends on these data is the compositional demand D, and it is the robustness of D that this panel examines. Fig 7b reports, for a representative set of species spanning three code types (NCBI tables 1, 11, and 12), the amino acid KLD (D) z-score of the SGC relative to the random-code ensemble under three successively richer definitions of the demand frequencies f_α: genome-derived (NCBI CDS, as in the original submission), proteome-level (UniProt reference

proteome), and proteome-level weighted by protein abundance (PaxDb). The arrows trace how each species' value shifts as the data source is enriched. These shifts are modest and occur in both directions, and in every case the SGC's demand divergence remains far below the random-code mean (all z-scores between roughly -2.1 and -3.4). The demand-matching signal is therefore robust to the data source: the abundance-weighted proteomic treatment suggested by the reviewer does not overturn our conclusion but corroborates it on more biologically faithful data.

We have added a citation to Radványi and Kun (2021) [40] in the Introduction (Lines 61–66) and discuss its relationship to our framework in the Discussion (Lines 636–639), where we note that our abundance-weighted, two-objective (E, D) analysis is complementary to their codon-usage-weighted robustness analysis.
