# Supplementary material for: The genetic code at the balance point of error and demand
Source: PLoS Comput Biol. 2026 Aug 11;22(8):e1014613. doi: 10.1371/journal.pcbi.1014613 (PMC13497292; doi:10.1371/journal.pcbi.1014613)
Supplement: Peer review history

# Peer review: Decision Letter 0

**DOI:** 10.1371/journal.pcbi.1014613.r001

**Authors:** Marc Robinson-Rechavi, Ilya Ioshikhes

17 Apr 2026

Balancing translational fidelity and amino acid demand in the evolution of the genetic code

PLOS Computational Biology

Dear Dr. Jo,

Thank you for submitting your manuscript to PLOS Computational Biology. After careful consideration, we feel that it has merit but does not fully meet PLOS Computational Biology's publication criteria as it currently stands. Therefore, we invite you to submit a revised version of the manuscript that addresses the points raised during the review process. It is especially important that you clarify the questions related to the fitness function, and the relation of your results to prior work.

Please submit your revised manuscript by Jun 17 2026 11:59PM. If you will need more time than this to complete your revisions, please reply to this message or contact the journal office at ploscompbiol@plos.org. When you're ready to submit your revision, log on to https://www.editorialmanager.com/pcompbiol/ and select the 'Submissions Needing Revision' folder to locate your manuscript file.

Please include the following items when submitting your revised manuscript:

* A letter that responds to each point raised by the editor and reviewer(s). You should upload this letter as a separate file labeled 'Response to Reviewers'. This file does not need to include responses to formatting updates and technical items listed in the 'Journal Requirements' section below.

* A marked-up copy of your manuscript that highlights changes made to the original version. You should upload this as a separate file labeled 'Revised Manuscript with Track Changes'.

* An unmarked version of your revised paper without tracked changes. You should upload this as a separate file labeled 'Manuscript'.

If you would like to make changes to your financial disclosure, competing interests statement, or data availability statement, please make these updates within the submission form at the time of resubmission. Guidelines for resubmitting your figure files are available below the reviewer comments at the end of this letter

We look forward to receiving your revised manuscript.

Kind regards,

Marc Robinson-Rechavi

Academic Editor

PLOS Computational Biology

Ilya Ioshikhes

Section Editor

PLOS Computational Biology

**Journal Requirements:**

1) We ask that a manuscript source file is provided at Revision. Please upload your manuscript file as a .doc, .docx, .rtf or .tex. If you are providing a .tex file, please upload it under the item type u2018LaTeX Source Fileu2019 and leave your .pdf version as the item type u2018Manuscriptu2019.

2) Please upload all main figures as separate Figure files in .tif or .eps format. For more information about how to convert and format your figure files please see our guidelines:

https://journals.plos.org/ploscompbiol/s/figures

3) Please amend your detailed Financial Disclosure statement. This is published with the article. It must therefore be completed in full sentences and contain the exact wording you wish to be published.

- State the initials, alongside each funding source, of each author to receive each grant. For example: "This work was supported by the National Institutes of Health (####### to AM; ###### to CJ) and the National Science Foundation (###### to AM)."

- State what role the funders took in the study. If the funders had no role in your study, please state: "The funders had no role in study design, data collection and analysis, decision to publish, or preparation of the manuscript.".

If you did not receive any funding for this study, please simply state: u201cThe authors received no specific funding for this work.u201d

**Reviewers' comments:**

Reviewer's Responses to Questions

**Comments to the Authors:**

**Please note here if the review is uploaded as an attachment.**

Reviewer #1: The paper under review deals with an exciting and yet contentious issue of the genetic code optimization level and the exact nature of evolutionary forces that shaped the code’s table. The main contributions of the paper are 1) taking account the existing frequencies of the amino acids to emphasize a multi-criteria nature of the code optimization; while in some of the previous studies this metric was already present (to the best of my knowledge, the amino acid frequencies first appeared in Ref. [34], it seems that this fact should be highlighted more in the text), in the current text the empirical proteomic demands are taken into account; 2) the mutation weights (Table 2) used in this study are finally given somewhat more careful treatment compared to previous analysis (but also see below); 3) a novel fitness function (Eq. 5 in the text) is suggested to consider the code evolution as multi-criteria optimization under two evolutionary forces: to minimize the cost of the possible errors and to guarantee that the code table has the sufficient capacity to produce the necessary amino acids in required amounts.

While I find the progress of the paper somewhat incremental I also think that the paper is carefully written, does a good job explaining the details of simulations, brings some relatively new ideas to the fields, and concludes with a non-surprising, and yet well defended conclusion that “[the standard genetic code] is a highly effective solution that balances fidelity against resource availability constraints.” Additionally, the paper fits well the scope of the journal, and therefore I recommend accepting the paper for publication. I do not have any major comments about the chosen approaches simply because almost all of them can be questioned and defended at the same time.

My only major remark is about the authors’ usage of the terms “mutations” and “mistranslations.” For instance, the term “transition” means exactly a point mutation of a certain kind and does not refer to the possible mistranslation at the same position during the translation process. The mutation weights chosen by Freeland and Hurst in 1998 refer specifically to the mutation probabilities, not to mistranslations. My understanding is that the authors tacitly assume that the frequencies of mutations are the same as the frequencies of mistranslations and use these terms interchangeably. Such a bold generalization requires at least some discussion and references to the known data on both mutation and mistranslation rates. I think that the paper will benefit from such a discussion.

Reviewer #2: This manuscript presents a sophisticated computational analysis of genetic code optimality using multi-objective framework. While the core methodology is reasonable and the findings are generally expected, the biological interpretation of the balancing parameter η and the circularity inherent in defining amino acid demand from coding sequences require additional scrutiny and validation. The authors should address the circularity concern by validating D with independent data sources, provide stronger biological justification for the fitness function form, and clarify the distinction between optimization algorithms and historical evolutionary processes.

My main question is whether the arbitrary fitness definition affect the patterns observed. Although weighted by the eta parameter, it is still arbitrary to assume a linear additive contribution to F for D and E.

The fitness function F = -E - ηD employs a crucial parameter η that modulates the relative weight of error load versus compositional demand. The authors calibrate η using standardized z-scores (0.56) based on the assumption that the SGC represents a balanced state between selective pressures. This approach risks circular reasoning, assuming the SGC is optimal to define the parameter that then "proves" it is optimal. Moreover, there is no independent biological justification for why natural selection would operate on z-score-normalized objectives rather than raw fitness costs.

The results in 442-448 (also 461-465) seems to be a mathematical consequence of the Fitness being the linear additive combination of D and E, not necessarily related to the property of codon code space.

Line 471. It is reasoned that the optimization of E without constraining D will lead to extremely low D with only one type of amino acid. However, D and E are statistically independent among random codes. Is this logically contradictory?

Line 82. The proteomic demand was derived from the codon usage frequency, or amino acid usage frequency?

Reviewer #3: The authors rightfully list some of the theories governing the evolution of the genetic code. A full review of them could be found, for example, in here: 10.1016/j.biosystems.2017.10.006. The two most important theories for the study at hand are the error minimization theory and the frozen accident theory. The importance of the first is obvious, but the second is also of importance. The authors wrote “This scenario suggests that the code’s evolution arrested, or “froze,” to preserve the functional integrity of protein synthesis”. The importance of the frozen accident theory is this, and not random assignment of codons and amino acids. Once there are many peptides and the cell relies on them (keep in mind that the genetic code evolved in an RNA world, so peptides gradually took over functions from ribozymes), changes to the genetic code is very detrimental. Experiments to change the genetic code proves that while it can be done, there is a considerable fitness cost to it. Consequently, I think (and I’m not the only one) that further optimization once it “froze” was not possible. The slight tweaking seen in alternative genetic codes mostly affects the STOP/Termination codons (thus do not disturb the amino acid composition of existing peptides). I would include a brief overview of the alternative genetic code in the introduction.

If we accept that at certain early point in the history of life on Earth, the SGC froze, then organisms a few billion years removed are poor proxy to study if the code is optimal. We can only use what we have, but at least we can try to find genomes that could be closer in composition to the one in which the SDG froze. One can posit that the SDG is rather optimal and find an environment in which it is so. It turns out that the SDG froze in a temperate environment (10.3390/life11080773).

Consequently, the choice of species in the analysis is rather strange. While the authors claim phylogenetic diversity, they also wanted to pick the different alternate genetic codes. A quick glance at the list of alternative tables shows that most comes from ciliates (Eukaryotes, Alveolata). An interesting clade in the Eukaryotic tree, but what it has to do with the origin of the genetic code is very questionable (understatement). Instead of using a bunch of Eukaryotes and some strange bacteria, I would suggest employing a good mix of bacteria and archaea. Recent phylogenies of bacteria (10.1126/science.abe0511) and archaea (10.1038/s41564-021-00918-8) could help in the picking.

Polar requirements is just one of the physicochemical properties usually used in assessing the mutational robustness of the genetic code. Why use this and not a mix of the usual ones, like hydropathy, molecular volume and isoelectric point (plus polar requirement)?

Furthermore, there are studies of mutational robustness that consider the composition of proteomes (such as 10.1038/s41598-021-90440-y). The authors should have a look at it.

Translational fidelity is mutations during the translation process. This process, as opposed to the replication fidelity (i.e. the fidelity of replicating DNA), is much more error prone. The replication error rate could fall into the realm employed by the author (10E-10 point mutations/base/generation) albeit for most of Eukaryotes used in the study it is 1-2 magnitudes higher. If the authors would like to study translation fidelity, which I think could be interesting, then using just the list of proteins is not the best option. There are proteins which are produced in higher quantity than others. Thus, errors in proteins translated more often weights more in the (translational) mutational load. For that, one would need transcriptome data. I’m aware that proteome is the best we can use now (albeit transcriptome data for some model organisms are available), I would suggest that you just call it “mutational fidelity” and not “translational fidelity”.

Because of the lack of clarity in what they would like to achieve, the poor choice of taxa and the lack of consideration for other physicochemical properties, I cannot suggest this manuscript for publication.

**********

**Have the authors made all data and (if applicable) computational code underlying the findings in their manuscript fully available?**

The PLOS Data policy requires authors to make all data and code underlying the findings described in their manuscript fully available without restriction, with rare exception (please refer to the Data Availability Statement in the manuscript PDF file). The data and code should be provided as part of the manuscript or its supporting information, or deposited to a public repository. For example, in addition to summary statistics, the data points behind means, medians and variance measures should be available. If there are restrictions on publicly sharing data or code —e.g. participant privacy or use of data from a third party—those must be specified.

Reviewer #1: Yes

Reviewer #2: Yes

Reviewer #3: Yes

**********

PLOS authors have the option to publish the peer review history of their article (what does this mean?). If published, this will include your full peer review and any attached files.

If you choose “no”, your identity will remain anonymous but your review may still be made public.

**Do you want your identity to be public for this peer review?**  For information about this choice, including consent withdrawal, please see our Privacy Policy.

Reviewer #1: No

Reviewer #2: No

Reviewer #3: **Yes:** Ádám Kun

[NOTE: If reviewer comments were submitted as an attachment file, they will be attached to this email and accessible via the submission site. Please log into your account, locate the manuscript record, and check for the action link "View Attachments". If this link does not appear, there are no attachment files.]

**Figure resubmission:**

**Reproducibility:**

?>

# Author comment: Author response to Decision Letter 1

**DOI:** 10.1371/journal.pcbi.1014613.r002

16 Jun 2026

Attachment Submitted filename: Response_to_reviewers.pdf

# Peer review: Decision Letter 1

**DOI:** 10.1371/journal.pcbi.1014613.r003

**Authors:** Marc Robinson-Rechavi, Ilya Ioshikhes

22 Jul 2026

Dear Dr. Jo,

We are pleased to inform you that your manuscript 'The genetic code at the balance point of error and demand' has been provisionally accepted for publication in PLOS Computational Biology.

Before your manuscript can be formally accepted you will need to complete some formatting changes, which you will receive in a follow up email. A member of our team will be in touch with a set of requests.

Please note that your manuscript will not be scheduled for publication until you have made the required changes, so a swift response is appreciated.

IMPORTANT: The editorial review process is now complete. PLOS will only permit corrections to spelling, formatting or significant scientific errors from this point onwards. Requests for major changes, or any which affect the scientific understanding of your work, will cause delays to the publication date of your manuscript.

Should you, your institution's press office or the journal office choose to press release your paper, you will automatically be opted out of early publication. We ask that you notify us now if you or your institution is planning to press release the article. All press must be co-ordinated with PLOS.

Thank you again for supporting Open Access publishing; we are looking forward to publishing your work in PLOS Computational Biology.

Best regards,

Marc Robinson-Rechavi

Academic Editor

PLOS Computational Biology

Ilya Ioshikhes

Section Editor

PLOS Computational Biology

***********************************************************

Reviewer's Responses to Questions

**Comments to the Authors:**

**Please note here if the review is uploaded as an attachment.**

Reviewer #1: The authors addressed my (quite limited) critique in the revision, I do not have any further comments, and I would like to recommend to accept the paper under review for publication.

Reviewer #3: The manuscript by Seo and co-workers has improved.

The analysis is still mainly about the effect of mistranslation of the 3rd position as that has a variable and high weight. But the discussion does not make it explicit that even local optimality could be very different if weights were different (which of course the results also show generally). Furthermore, can you give an example of a random code? The method on code randomization is vague. Where are the cut points? Each triplet has 9 neighbours, so the blocks can form in very different ways. Does the generated random codes also from block with wobbling 3rd positions? If so, just state it more clearly. If not, then it is not surprising that the SGC and variants are that much more optimal: the model assumes that 3rd positions wobble and can change a lot. Would AGA, AGC, AAA, AAC be a continuous block? In the SGC block are formed with the same 3rd position or having a block with only half or one-fourth of such block. But there is no code with an amino acid occupying two half blocks (like in my examples). This is the reason why most code table randomization retains the block structure but reassigns the amino acids. I do not suggest that the exact block structure of the SGC (or variants) need to be employed, but the random assignment should have a plausible block structure.

P2L47 “provided an initial bias, setting the chemical boundaries for early, non-enzymatic translation systems” I do not think that there was ever non-enzymatic translation. The stereochemical hypothesis offers an idea for the initial assignment of amino acids to (proto)tRNA not necessarily for translation.

P16L541: If codon reassignment always has a fitness cost, then this particular outcome is derived from not considering STOP codons. For example, if the UGA STOP codon is replaced with tryptophane (quite common change), it will increase translational fidelity. Similarly, increasing the number of methionine codons to 2 also helps (present in about 5 alternate tables). I think you have omitted STOP codons from your analysis. It is fine. But then also note why some alternate codes are not as good as they could be.

P19: The authors argue that mistranslation is more important than genetic mutation for pressure of fidelity. This might be so. But proteomes, as analysed here, are not the best proxies for that. For genetic fidelity that would be great. For transcription/translation fidelity transcriptome analysis with abundances would be necessary. Please note this in the discussion.

Minor comments

Figure 3. Can you use different line widths so that the figure is easy to read even when printed black and white?

P17L553 The Gracilibacteria now have a valid name Minisyncoccota. Just write “Candidatus Gracilibacteria (Minisyncoccota)”. See the List of Prokaryotic names with Standing in Nomenclature: https://lpsn.dsmz.de/phylum/minisyncoccota

**********

**Have the authors made all data and (if applicable) computational code underlying the findings in their manuscript fully available?**

The PLOS Data policy requires authors to make all data and code underlying the findings described in their manuscript fully available without restriction, with rare exception (please refer to the Data Availability Statement in the manuscript PDF file). The data and code should be provided as part of the manuscript or its supporting information, or deposited to a public repository. For example, in addition to summary statistics, the data points behind means, medians and variance measures should be available. If there are restrictions on publicly sharing data or code —e.g. participant privacy or use of data from a third party—those must be specified.

Reviewer #1: Yes

Reviewer #3: Yes

**********

PLOS authors have the option to publish the peer review history of their article (what does this mean?). If published, this will include your full peer review and any attached files.

If you choose “no”, your identity will remain anonymous but your review may still be made public.

**Do you want your identity to be public for this peer review?**  For information about this choice, including consent withdrawal, please see our Privacy Policy.

Reviewer #1: No

Reviewer #3: **Yes:** Ádám Kun

# Editor report: Acceptance letter

**DOI:** 10.1371/journal.pcbi.1014613.r004

**Authors:** Marc Robinson-Rechavi, Ilya Ioshikhes

PCOMPBIOL-D-26-00560R1

The genetic code at the balance point of error and demand

Dear Dr Jo,

I am pleased to inform you that your manuscript has been formally accepted for publication in PLOS Computational Biology. Your manuscript is now with our production department and you will be notified of the publication date in due course.

The corresponding author will soon be receiving a typeset proof for review, to ensure errors have not been introduced during production. Please review the PDF proof of your manuscript carefully, as this is the last chance to correct any errors. Please note that major changes, or those which affect the scientific understanding of the work, will likely cause delays to the publication date of your manuscript.

Soon after your final files are uploaded, unless you have opted out, the early version of your manuscript will be published online. The date of the early version will be your article's publication date. The final article will be published to the same URL, and all versions of the paper will be accessible to readers.

For Research, Software, and Methods articles, you will receive an invoice from PLOS for your publication fee after your manuscript has reached the completed accept phase. If you receive an email requesting payment before acceptance or for any other service, this may be a phishing scheme. Learn how to identify phishing emails and protect your accounts at https://explore.plos.org/phishing.

Thank you again for supporting PLOS Computational Biology and open-access publishing. We are looking forward to publishing your work!

With kind regards,

Kannan R K Kuppusamy, B.TECH BIOTECHNOLOGY

PLOS Computational Biology | Carlyle House, Carlyle Road, Cambridge CB4 3DN | United Kingdom ploscompbiol@plos.org | Phone +44 (0) 1223-442824 | ploscompbiol.org | @PLOSCompBiol
